# CIB2 regulates mTORC1 signaling and is essential for autophagy and visual function

**DOI:** 10.1038/s41467-021-24056-1

**Published:** 2021-06-23

**Authors:** Saumil Sethna, Patrick A. Scott, Arnaud P. J. Giese, Todd Duncan, Xiaoying Jian, Sheikh Riazuddin, Paul A. Randazzo, T. Michael Redmond, Steven L. Bernstein, Saima Riazuddin, Zubair M. Ahmed

**Affiliations:** 1 Department of Otorhinolaryngology - Head & Neck Surgery, University of Maryland School of Medicine, Baltimore, MD USA; 2 Department of Ophthalmology & Visual Sciences, University of Louisville, Louisville, KY USA; 3 Laboratory of Retinal Cell and Molecular Biology, National Eye Institute, National Institutes of Health, Bethesda, MD USA; 4 Laboratory of Cellular and Molecular Biology, National Cancer Institute, National Institutes of Health, Bethesda, MD USA; 5 Allama Iqbal Medical College, University of Health Sciences, Lahore, Pakistan; 6 Department of Ophthalmology and Visual Sciences, University of Maryland School of Medicine, Baltimore, MD USA

**Keywords:** Autophagy, Mechanisms of disease, Molecular medicine

## Abstract

Age-related macular degeneration (AMD) is a multifactorial neurodegenerative disorder. Although molecular mechanisms remain elusive, deficits in autophagy have been associated with AMD. Here we show that deficiency of calcium and integrin binding protein 2 (CIB2) in mice, leads to age-related pathologies, including sub-retinal pigment epithelium (RPE) deposits, marked accumulation of drusen markers APOE, C3, Aβ, and esterified cholesterol, and impaired visual function, which can be rescued using exogenous retinoids. *Cib2* mutant mice exhibit reduced lysosomal capacity and autophagic clearance, and increased mTORC1 signaling—a negative regulator of autophagy. We observe concordant molecular deficits in dry-AMD RPE/choroid post-mortem human tissues. Mechanistically, CIB2 negatively regulates mTORC1 by preferentially binding to ‘nucleotide empty’ or inactive GDP-loaded Rheb. Upregulated mTORC1 signaling has been implicated in lymphangioleiomyomatosis (LAM) cancer. Over-expressing CIB2 in LAM patient-derived fibroblasts downregulates hyperactive mTORC1 signaling. Thus, our findings have significant implications for treatment of AMD and other mTORC1 hyperactivity-associated disorders.

## Abstract (web summary)

Age-related macular degeneration (AMD) has been connected to deficits in autophagy. Here, the authors demonstrate, in mice and dry-AMD patient samples, that calcium and integrin binding protein 2 (CIB2) regulates Rheb-mTORC1 signaling axis, and subsequently autophagy.

## Introduction

Rod and cone photoreceptors (PR), are highly specialized neurons, and serve as the site of signal transduction where irradiant light is converted into neuronal signals in the eye. PR outer segments (OS) are dedicated to phototransduction, however, they depend heavily on support from the retinal pigment epithelium (RPE), a single-layer of polarized cobblestone-shaped post-mitotic cells. RPE has multiple functions essential to normal vision, such as absorbing excess light, regenerating vitamin A-derived chromophores, and shuttling nutrients and metabolites from the sub-retinal space to the PR^1^. RPE supports the PR and hence vision by fulfilling several critical functions, including non-canonical autophagy or LC3-associated phagocytosis (LAP) of PR outer segments^1,2^. Likewise, macroautophagy (henceforth autophagy) is a catabolic process, which removes cellular debris, damaged/aged organelles, and shares many features with LAP. Autophagy is an essential process for all cells, but its function is especially notable in post-mitotic cells such as the RPE^2^ that have the highest life-long phagocytic load of any cell-type in the body.

Growing evidence suggests that RPE dysfunction, specifically in OS digestion and clearance, leads to age-related photoreceptor dysfunction without gross retinal degeneration^2–4^. For instance, RPE-specific deletion of ATG5, caveolin-1, or the β-crystallin protein leads to the impaired digestion of OS but does not cause gross retinal degeneration. However, the PR function (measured by electroretinography (ERG)) is substantially impaired^2–4^. Further, phagocytosis/ autophagy defects have been implicated in the dry form of age-related macular degeneration (AMD), a progressive degenerative disease of the macula, a specialized region of the retina responsible for daytime vision^5^. AMD often causes central vision loss and irreversible blindness, and affects 10% of the population aged 65-75 years and 25% of those aged ≥75 years. By 2050, its prevalence is expected to increase by 50%^6,7^. AMD is categorized as either “wet” (associated with choroidal neovascularization) or “dry” (associated with atrophy and progressive thinning of retinal layers). Dry AMD occurs in ~90% of the cases and can cause permanent vision loss if left untreated^8^.

Besides AMD, deficits in autophagy are linked to lifespan and several diseases such as Alzheimer’s, Parkinson’s, certain cancers, and metabolic disorders amongst others^9–11^. The mechanistic target of rapamycin complex 1 (mTORC1) is a well-established negative regulator of autophagy. mTORC1 also integrates nutrient and growth factors via its nutrient-sensing arm and tuberous sclerosis complex (TSC) complex, respectively^12,13^. TSC complex (consisting of TSC1, TSC2, and TBC1D7) directly regulates the small GTPase, Rheb (Ras homolog enriched in brain)^14^. GTP-bound Rheb is a potent and obligate activator of mTORC1 that acts via allosterically realigning the kinase active site of mTORC1^15^. Aberrant mTORC1 signaling is also implicated in aging, cancers, obesity, and Alzheimer’s amongst others^16^. Therefore, deciphering the molecular regulators of autophagy and the mTORC1 pathway has significant ramifications for the fundamental cellular aging process and the etiology of autophagy-related disorders.

We previously identified that CIB2 impairment was associated with deafness and/or vision deficits in humans, zebrafish, and drosophila^17^. Downregulation of *cib2* in drosophila significantly reduces photoresponse amplitudes and causes light-dependent retinal degeneration^17^. However, the molecular mechanism of such CIB2-associated vision loss remains unknown. Here, we show that the ablation of CIB2, specifically in the RPE, causes an age-related phenotype in mice and dysregulation of phagolysosomal processing of OS. Further, RPE-specific ablation of CIB2 leads to dysregulation of mTORC1 and autophagy. Our interaction studies reveal the preferential binding of CIB2 to the inactive state of Rheb, which then acts as a negative regulator of mTORC1.

## Results

### Loss of CIB2 leads to age-related PR dysfunction and RPE pathology

**Fig. 1:**
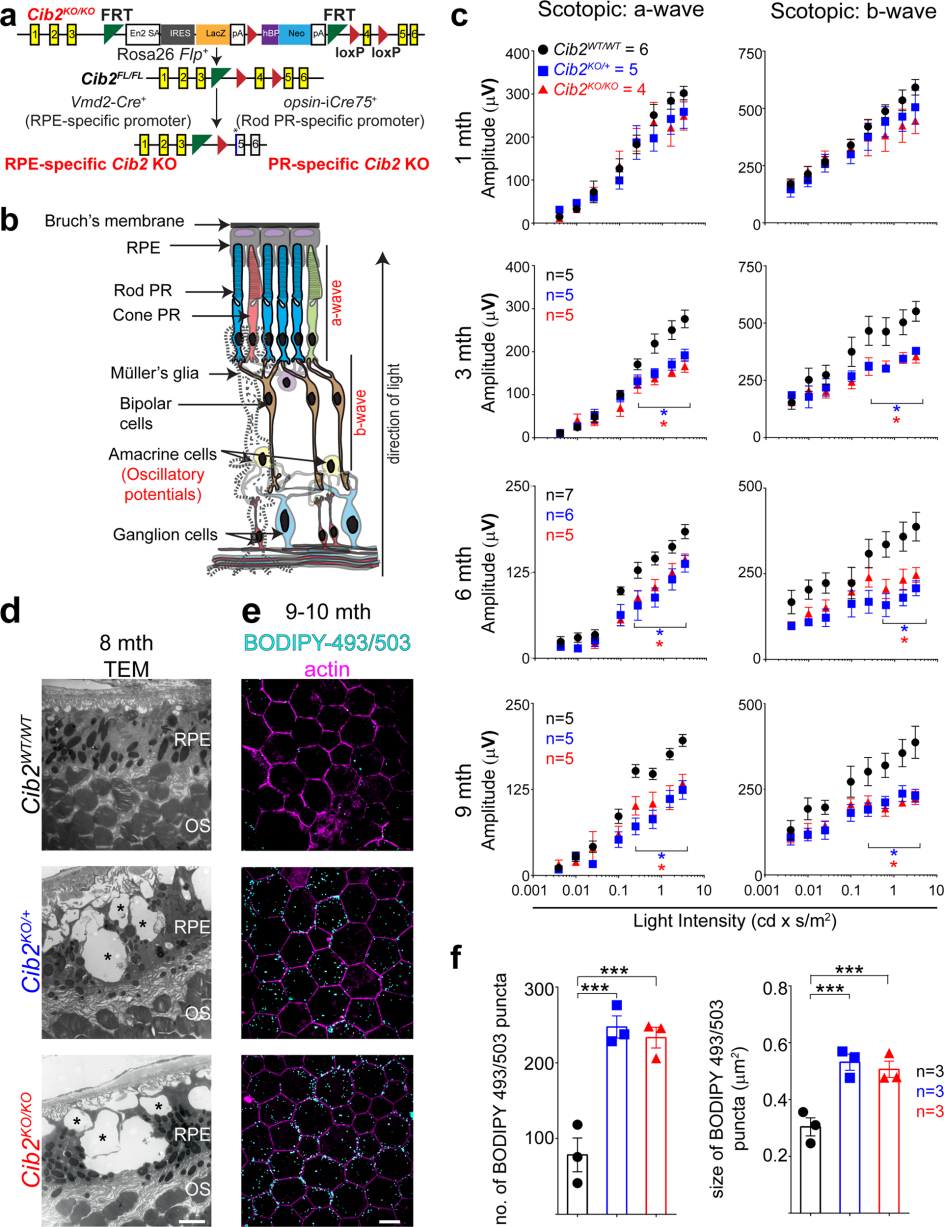
Loss of CIB2 leads to age-related photoreceptor dysfunction and RPE pathophysiology. **a** Schematic of specific constructs used to generate mouse models. **b** Schematic representation of the multiple retinal layers. ERG a-wave originates from photoreceptors (PR), while b-wave amplitude originates from Müller glia and bipolar cells, and oscillatory potentials come from the amacrine cells. RPE retinal pigment epithelium. **c** Quantification of scotopic responses from WT (*Cib2*^*WT/WT*^), *Cib2*^*KO/+*^, and *Cib2*^*KO/KO*^ mice at indicated ages reveals progressive loss of both a- (left panels) and b-wave (right panels) amplitudes in *Cib2*-deficient mice. **d** TEM micrographs of RPE/ outer segment (OS) interface from 8-months-old mice revealed vacuoles (*) and basal infoldings loss specifically in *Cib2*^*KO/+*^ and *Cib2*^*KO/KO*^ mutants (*n* = 3 per genotype). Scale bar, 2 μm. **e** Representative photomicrographs of RPE whole mounts from 9-to-10-months-old mice for denoted genotype showing accumulation of neutral lipid marker BODIPY-493/503 (cyan) in *Cib2*-deficient mice. Phalloidin (magenta) was used to decorate the actin cytoskeleton. Scale bar, 10 µm. **f** Quantification of average number (left) or average size (right) of BODIPY-493/503-stained puncta per mouse shown in **e**. At least three images per mouse (~25 cells/image) were quantified and averages per mouse are shown for WT, *Cib2*^*KO/+*^, and *Cib2*^*KO/KO*^ mice (*n* = 3 each). Data presented as mean ± SEM; each data point represents an individual animal. One-way ANOVA and Bonferroni post hoc test, *p* < 0.05 (*) or <0.001 (***).

Within the retina, CIB2 is expressed in the RPE, PRs, and certain ganglion cells (Supplementary Fig. 1a–g). To determine the exact function of CIB2 in the mammalian retina, we used *Cib2*^*tm1a*^ mutant mice (Fig. 1a, *Cib2*^*KO*^ henceforth)^18^ that lack CIB2 in the RPE (Supplementary Fig. 1f) as well as other retinal layers. Non-invasive in vivo scotopic full-field ERGs (illustrated in Fig. 1b), which preferentially analyze rod PR function, revealed no difference in a-wave (derived primarily from the photoreceptor layer) or b-wave (derived from the inner retina, predominantly Müller and outer nuclear bipolar cells) amplitudes in 1-month-old *Cib2*-deficient mice. However, at 3, 6, and 9 months, both *Cib2*^*KO/+*^ and *Cib2*^*KO/KO*^ mice exhibited similar age-related declines (~20–30%) in both a- and b-wave amplitudes, as compared with those of wild type (WT) mice (Fig. 1c). However, the declines were not demonstrated in latency or oscillatory potential, suggesting the inner retinal function was not impacted (Supplementary Fig. 2a, c). In contrast, the b-wave amplitude of the photopic ERG, which evaluates cone PRs, was similar across all three genotypes (Supplementary Fig. 2b). These results suggest that both haploinsufficiency and complete loss of CIB2 leads to rod PR dysfunction in mice.

To determine whether corresponding anatomical changes occurred with progressive loss of PR function, we evaluated the morphology of the retina in 2 and 8- to 9-months-old *Cib2*-deficient mice. Light microscopy-based morphometric analysis of the specific retinal strata revealed no significant differences in *Cib2*^*KO/+*^ and *Cib2*^*KO/KO*^ compared to those of WT mice at either age (Supplementary Fig. 2d, e). However, at the transmission electron microscopic (TEM) level, we observed vacuoles with undigested membranous material and loss of basal infoldings in the RPE of *Cib2*^*KO/+*^ and *Cib2*^*KO/KO*^ mice, but not in age-matched WT mice (Fig. 1d). Staining with the neutral lipid tracer BODIPY^TM^ 493/503 revealed twofold more lipid droplets in the RPE of aged *Cib2*^*KO/+*^ and *Cib2*^*KO/KO*^ mice (Fig. 1e, f). Together, these results suggest that loss of CIB2 causes excessive lipid accumulation in RPE and is associated with progressive rod PR dysfunction.

### Selective ablation of CIB2 in RPE but not in PRs recapitulates retinal pathophysiology

**Fig. 2:**
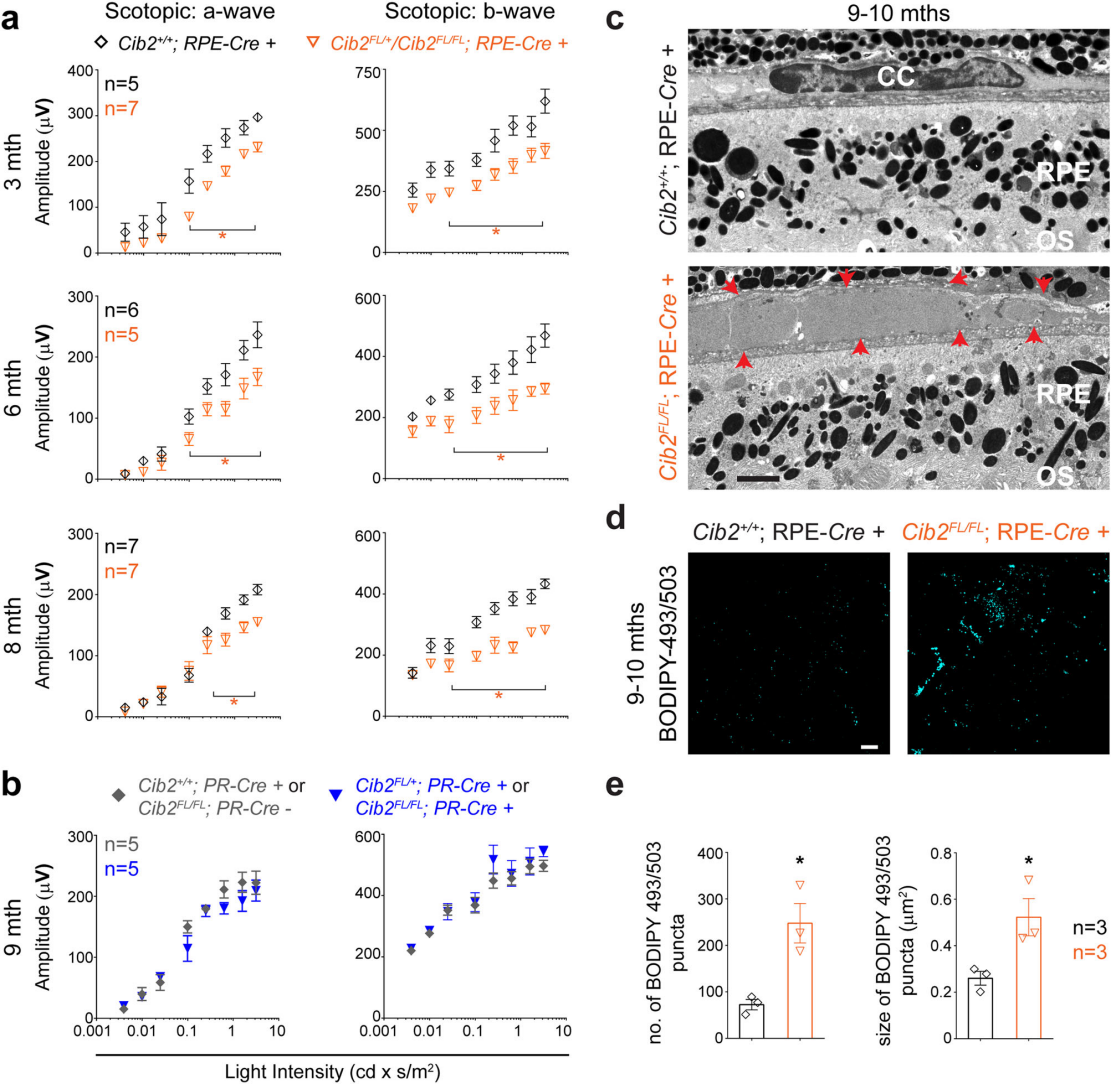
Loss of CIB2 specifically from RPE, but not rod photoreceptors recapitulates age-related phenotype. **a**, **b** Quantification of scotopic a- (left panels) and b-wave (right panels) amplitudes for RPE-*Cre+* (**a**) and PR-*Cre* mice (**b**) at indicated ages. Loss of CIB2 specifically from RPE but not rod photoreceptors (PR) resulted in ERG deficits. **c** TEM micrographs of 9-to-10-months-old RPE-*Cre* +; *Cib2*^*+/+*^ (control, top panel) and *Cib2*^*FL/FL*^ (bottom panel) mice (*n* = 3 per genotype). Red arrows (bottom panel) indicate boundaries of deposits present between RPE and choroid tissue in RPE-specific *Cib2*-mutant mice. Scale bar, 2 μm. RPE retinal pigment epithelium, OS outer segment, CC choriocapillaris. **d** Representative photomicrographs of RPE whole mounts from 9-to-10-months-old mice for denoted genotype demonstrate accumulation of neutral lipids in RPE-specific *Cib2*-mutant mice. Scale bar, 10 µm. **e** Quantification of average number (left) or size (right) of BODIPY-493/503 puncta per mouse shown in **d**. At least three images per mouse (~25 cells/image) were quantified and averages per mouse are shown for denoted genotypes (*n* = 3 per genotype). Data presented as mean ± SEM. Each data point represents an individual mouse. Unpaired two-tailed *t*-test, *p* < 0.05 (*).

In the mammalian retina, PRs and RPE are codependent, therefore, the functional impairment of one layer negatively impacts the other^2–4,19^. To pinpoint the anatomical site of age-related rod PR dysfunction seen in global *Cib2*-deficient mice, we generated two cell type-specific mutant strains, in which *Cib2* expression was ablated either in rod PR (designated PR-*Cre+*) using the rod PR-specific *rhodopsin*–*iCre75*^20^, or in the RPE (designated RPE-*Cre+*) using the RPE-specific *Vmd2* promoter^21^ (Fig. 1a and “Methods” section). ERG analyses revealed that the RPE-specific *Cib2*^*KO*^ (*Cib2*^*flox/flox*^*;* RPE-*Cre+* and *Cib2*^*flox/+*^*;* RPE-*Cre+*) mice had reduced ERG amplitudes compared to the control mice (*Cib2*^*+/+*^; RPE-*Cre+*) as early as 3 months of age. Similar to *Cib2* global knockout mice, ERG amplitudes declined even further with age (Fig. 2a). In contrast, we found no differences in either a- or b-wave amplitudes with PR-specific *Cib2*^*KO*^ (*Cib2*^*flox/flox*^*;* PR-*Cre+* and *Cib2*^*flox/+*^*;* PR-*Cre+*) mice compared to control mice at any tested age (Fig. 2b and Supplementary Fig. 3a, b). Taken together, the retinal functional and morphological analyses in three mutant strains suggest that rod PR dysfunction occurs secondary to the loss of CIB2 function in RPE but not in rod PRs.

**Fig. 3:**
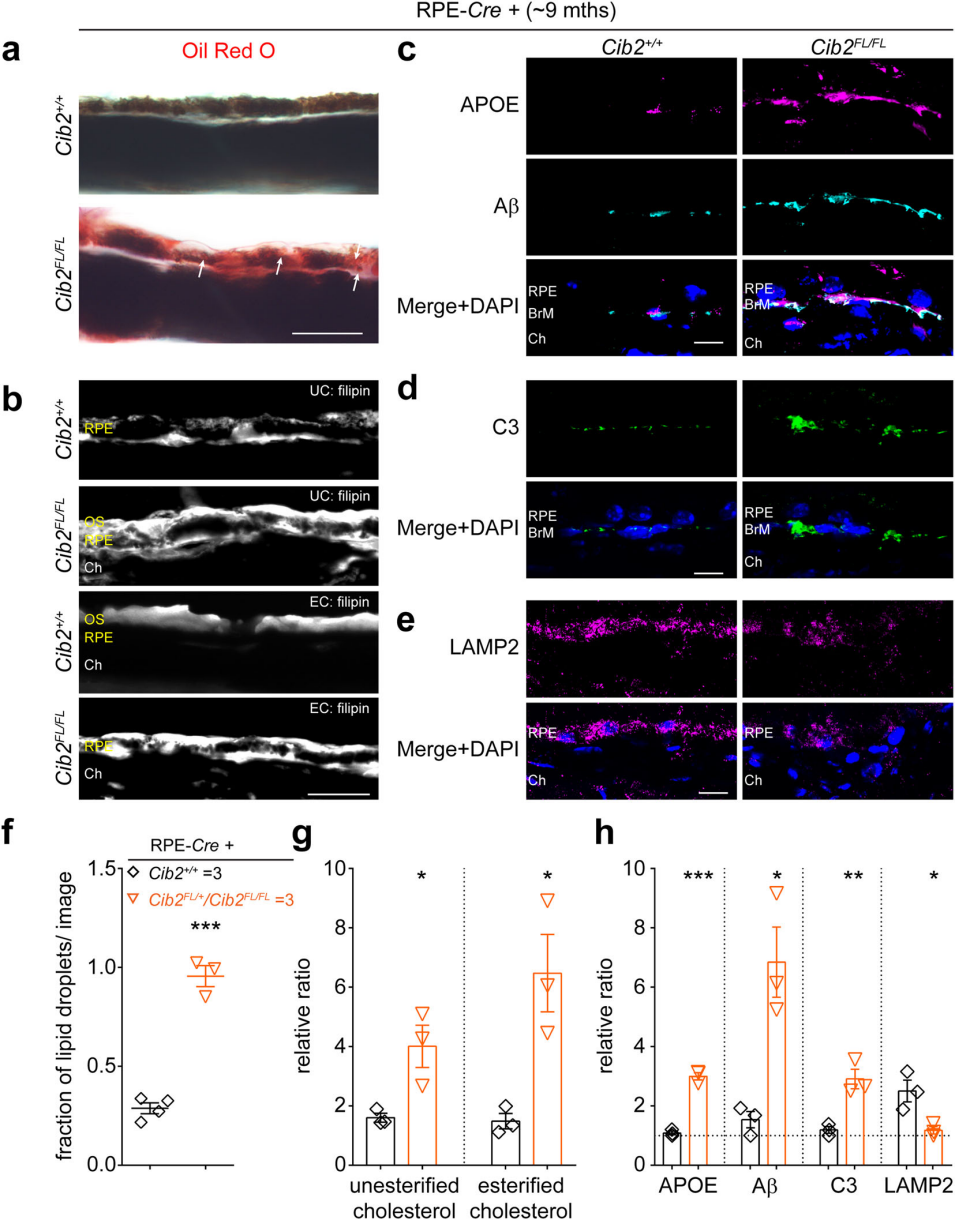
*Cib2*-mutant mice mimic the drusen pathologies reported in human RPE with dry AMD. **a** Oil red O staining shows a marked accumulation of lipids within and below the RPE in mutant mice. Arrows indicate lipid droplets. **b** Filipin staining for unesterified cholesterol (UC, top two panels) and esterified cholesterol (EC, bottom two panels) shows the marked accumulation of both UC and EC, particularly in mutant mice, below the RPE. **c**, **d** Confocal micrographs of cryosections of 9-to-10-months-old mice of denoted genotype shows the accumulation of specific proteins APOE, Aβ (**a**), and C3 (**b**) below the RPE, particularly in mutant mice. **e** Confocal micrographs of cryosections of 9-to-10-months-old mice of denoted genotype show that the levels of lysosomal protein LAMP2 are lower within the RPE of mutant mice. Scale bar, 10 μm (**a**, **b**), 20 μm (**c**–**e**). RPE retinal pigment epithelium; BrM Bruch’s membrane; Ch choroid; OS outer segments. **f** Quantification for fraction of lipid droplets for images presented in **a**. **g** Quantification of the relative ratio of denoted lipid species for images shown in **b**. **h** Quantification of the relative ratio of denoted proteins levels for images shown in **c**–**e**. At least three images per mouse were quantified and averages per mouse are shown for denoted genotypes (*n* = 3 per genotype). Data presented as mean ± SEM. Each data point represents an individual mouse. Unpaired two-tailed *t*-test, *p* < 0.05 (*), <0.01 (**), or <0.001 (***).

With respect to the thickness of RPE- and PR-specific *Cib2*^*KO*^ mice retinal strata, we found no morphometric differences (Supplementary Fig. 3c, d), however, TEM revealed sub-RPE deposits in RPE-specific *Cib2*^*KO*^ mice (Fig. 2c) that were absent in PR-specific mutants (Supplementary Fig. 3e). As seen in *Cib2* global knockout mice, we also found the presence of an increased amount of lipid droplets in the RPE of RPE-specific *Cib2*^*KO*^ mice (Fig. 2d, e). We further confirmed the presence of these lipid droplets in 9- to 10-months-old RPE-specific mutant mice by staining frozen transverse sections with Oil Red O (Fig. 3a). Lastly, we also found increased filipin staining specifically under RPE, which revealed the accumulation of both unesterified and esterified cholesterol (Fig. 3b). Esterified cholesterol, in particular, has been shown to accumulate in drusen and deposits in the Bruch’s membrane in humans suffering with dry AMD^22^.

### Molecular characterization of sub-RPE deposits in *Cib2*-deficient mice

As presented in humans, AMD is a progressive, age-related, and degenerative disease of the retina. AMD is categorized as either wet or dry, due to either choroidal neovascularization or progressive thinning of macula layers, respectively^23^. Sub-RPE drusen are a prominent feature observed in humans suffering with dry AMD^24^. Protein-level analysis of drusen found in dry AMD patients, revealed accumulation of proteins such as apolipoprotein E (APOE)^25^, β-Amyloid (aβ), and complement factor 3 (C3), a genetically validated AMD risk factor^26–28^. Meanwhile, a recent study showcased that the levels of LAMP2/CD107b (a lysosomal membrane protein) are reduced in AMD RPE/choroid tissues^29^. Observation of similar sub-RPE deposits in TEM micrographs in *Cib2*-mutant mice prompted us to investigate the molecular composition of the deposits. We found that in 9-to-10-months-old RPE-specific *Cib2* mutant mice there was a marked accumulation of APOE, β-Amyloid (aβ), and C3 (Fig. 3c, d) below the RPE and reduced expression of LAMP2/CD107b within the RPE (Fig. 3e). Ultimately, our data shows that bonafide AMD drusen markers and lipid species are enriched in the sub-RPE deposits in *Cib2-*mutant mice, alluding to the potential role of CIB2 in human AMD.

### Loss of CIB2 leads to dysregulated clearance of photoreceptor outer segments

**Fig. 4:**
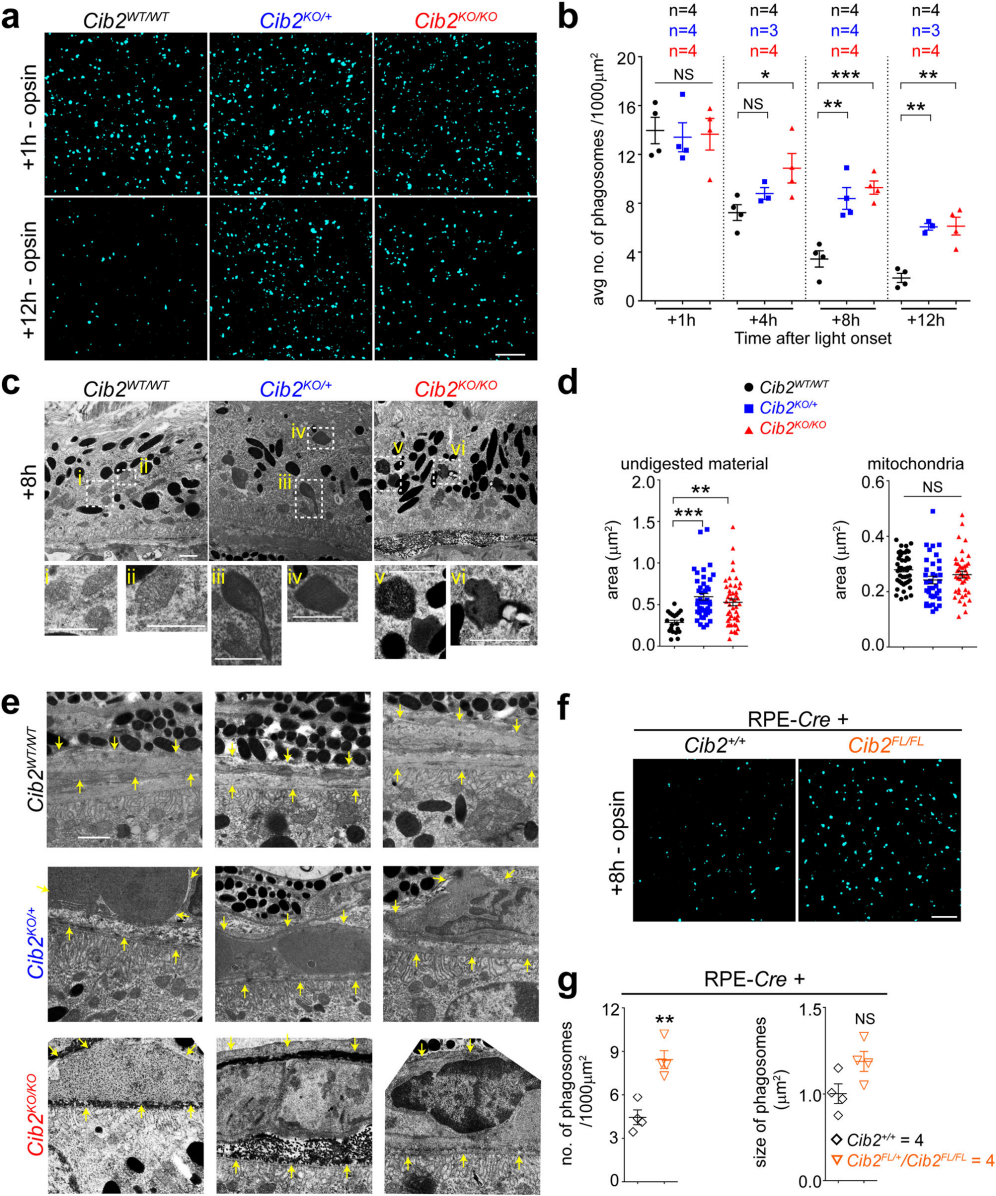
Deficiency of CIB2 resulted in impaired phagolysosomal processing of OS. **a** RPE whole mounts from 3- to 4-months-old mice euthanized at denoted times after light onset and immunostained with opsin Ret-P1 antibody (cyan) shows initial binding and ingestion of opsin-phagosomes is similar across genotypes (top panels), however, the phagolysosomal digestion is slower in mutant mice (bottom panels) as compared to the WT control. Scale bar, 10 µm. **b** Quantification of average number of opsin-phagosomes shown in **a** and Supplementary Fig. 4a. **c** TEM micrographs of 2-months-old mice euthanized 8 h after light onset show accumulation of undigested material, and fused remnants of phago-melanosomes in mutant mice. **i**–**vi** Insets show magnified images of normal phagosomes in WT mice and improperly processed phagosomes only in *Cib2*^*KO/+*^ and *Cib2*^*KO/KO*^ mice. Scale bar, 1 μm. **d** Quantification of individual phagosomal (left) and mitochondrial (right) areas (as control) for images shown in **c** further confirms marked accumulation of phagosomal area in mutant mice. All phagosomes and 15 mitochondria/image in 3–5 images per mouse were counted (*n* = 3 per genotype). **e** TEM micrographs from 2-months-old mice show marked thickening and accumulation of debris under the RPE in mutant mice. Yellow arrows indicate the boundary between the basal surface of RPE and choroid. Scale bar, 1 μm. **f** Representative RPE whole mount images from 3-to-4-months-old mice euthanized 8 h after light onset and immunostained with opsin Ret-P1 antibody (cyan) show slower phagolysosomal clearance, quantified in **g**, in RPE-specific *Cib2*-mutant mice. Scale bar, 10 µm. At least three images per mouse (~30 cells/ image) were quantified and the average per animal is shown for denoted genotypes. Data presented as mean ± SEM. Each data point represents an average per individual mouse unless specified. One-way ANOVA and Bonferroni post hoc test (**b**, **d**) or unpaired two-tailed *t-*test (**g**), *p* < 0.05 (*), <0.01 (**), or <0.001 (***), NS not significant.

Defects in the OS renewal process in mice can lead to the formation of vacuoles and an accumulation of lipids^4^, a phenotype similar to that presented in *Cib2*^*KO*^ mice. We reasoned that a lack of CIB2 may lead to debris and vacuole formation in older mice (8–10 months) due to faulty phagolysosomal processing of the OS by the RPE early in life (2–4 months). We took advantage of the characteristic diurnal circadian nature of RPE-mediated OS phagocytosis to quantify engulfment (early after light onset) and digestion (rest of the day), by in situ quantification of phagosomes within the RPE, at specific times after light onset. The OS are composed of 50% lipids and 50% proteins, and rhodopsin constitutes about 80% of rod OS protein content. Thus, any rhodopsin found in the RPE is from phagocytosed OS and can be used as a marker to track the OS renewal process^3,30^. In situ, we found that counts of opsin-phagosomes in whole mounts of RPE/choroid 1 h after light onset, corresponding to the peak of OS engulfment, were similar in *Cib2*^*KO*^ and WT mice, indicating that binding and engulfment of OS were unaffected by *Cib2* deficiency (Fig. 4a, top panel and b). However, *Cib2*^*KO*^ mice exhibited significantly higher numbers of opsin-phagosomes eight and twelve hours following the onset of light, representing slower phagolysosomal clearance (Fig. 4a, bottom panel and b and Supplementary Fig. 4a, b). The average diameters of opsin-phagosomes decreased in tandem with time from the onset of light-on in WT animals. On the other hand, the decreases in *Cib2*^*KO*^ mice were delayed, further confirming that *Cib2* deficiency leads to phagolysosomal dysfunction (Supplementary Fig. 4c). TEM imaging of 2-months-old mice retinae that were dissected eight hours after light onset revealed that *Cib2*^*KO/+*^ and *Cib2*^*KO/KO*^ mice exhibited marked accumulation of improperly-digested remnants, lipid droplets fused with undigested material, and/or fused phago-melanosomes (Fig. 4c, d). Similar deficits were observed in RPE-specific *Cib2*^*KO*^ mice 8 h after light onset (Fig. 4e–g).

**Fig. 5:**
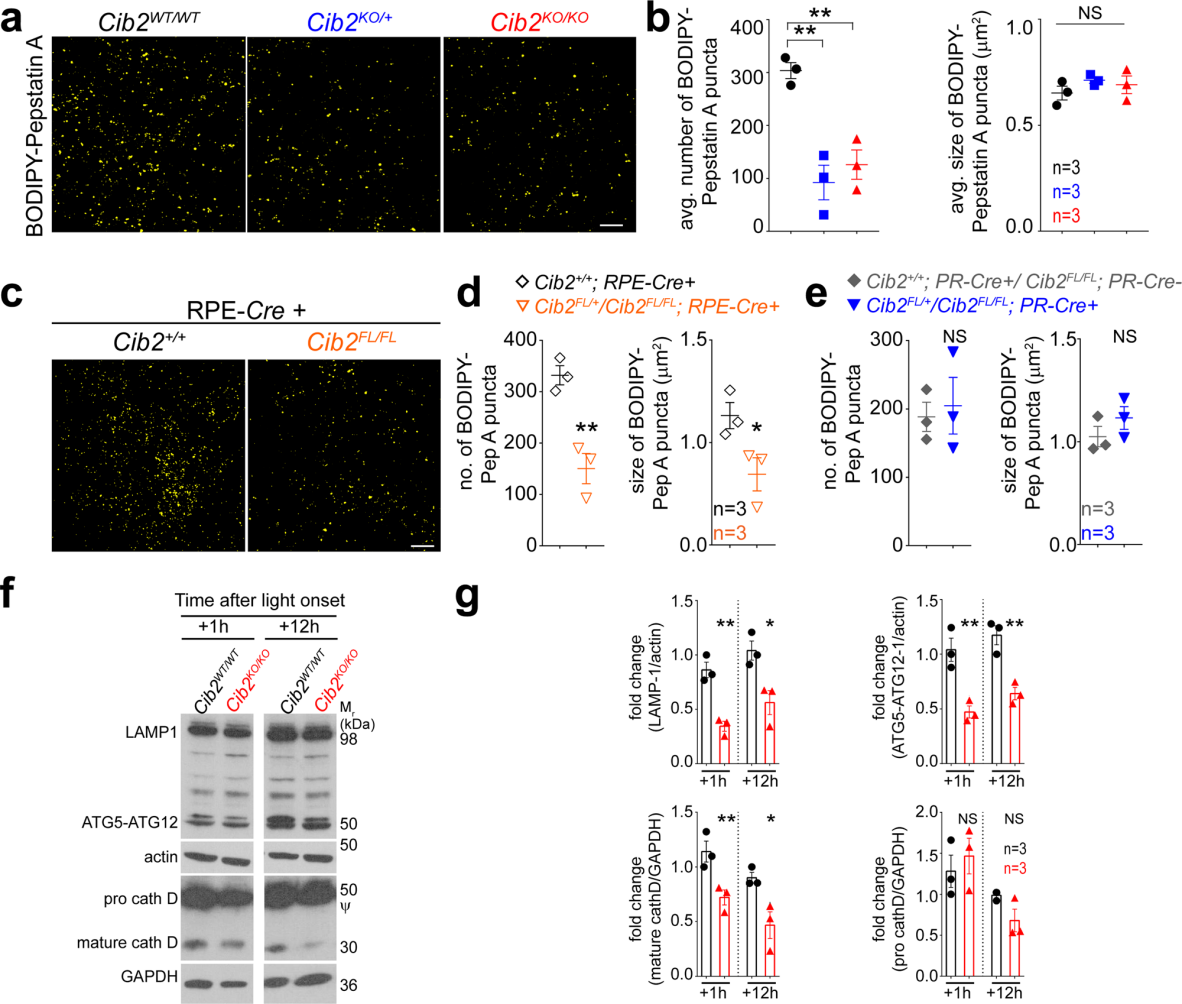
Deficiency of CIB2 resulted in impaired lysosomal machinery. **a** RPE whole mounts from 3-to-4-months-old mice stained with BODIPY-pepstatin A, a dye which binds to active cathepsin D only, demonstrate fewer indirectly stained lysosomes, quantified in **b**, in *Cib2*-deficient mice. RPE distinguished by phalloidin staining is shown in Supplementary Fig. 5a. Scale bar, 10 µm. **c** Similarly, RPE whole mounts from 3-to-4-months-old mice show that RPE-specific *Cib2* mutants have less BODIPY-pepstatin A stained lysosomes. RPE distinguished by phalloidin staining are shown in Supplementary Fig. 5b. Scale bar, 10 µm. **d**, **e** Quantification of average numbers (left) or sizes (right) of BODIPY-pepstatin A puncta per mouse in RPE-*Cre+* (**d**) and PR-*Cre* (**e**) mice shown in panel **c** and Supplementary Fig. 5b. **f**, **g** Representative immunoblots (**f**) for denoted proteins from RPE/choroid lysates obtained from mice euthanized either 1 or 12 h after light onset for denoted genotypes indicates that specified lysosomal/autophagy protein levels are decreased in mutant mice at both time points assessed. *ψ*, pro-cathepsin D immunoblot is overexposed for mature-cathepsin D to be observable, quantified in **g**. *n* = 3/genotype/time point. Pro-cathepsin D levels were quantified from non-saturated exposures. At least three images per mouse (~30 cells/image) were quantified and the average per animal is shown for denoted genotypes. Data presented as mean ± SEM. Each data point represents an average per individual mouse unless specified. One-way ANOVA and Bonferroni post hoc test (**b**) or unpaired two-tailed *t*-test (**d**, **e**, **g**), *p* < 0.05 (*) or <0.01 (**), NS not significant.

Proper clearance of ingested OS requires optimally functioning lysosomal machinery. In the RPE, the aspartyl protease cathepsin D is essential for phagosome digestion^3,31–33^. Cathepsin D is translated as ~50 kD pro-cathepsin D and matures to its active form (~30 kDa) in the low-pH lysosomal milieu. RPE flat mounts ex vivo probed with BODIPY-pepstatin A, which binds specifically to mature-cathepsin D, showed a marked reduction of about 2.5 fold in indirectly stained lysosomes, and hence available mature-cathepsin D in *Cib2*^*KO/+*^, *Cib2*^*KO/KO*^, and RPE-specific *Cib2*^*KO*^ mice (Fig. 5a–d and Supplementary Fig. 5a, 5b, left panel), but not in PR-specific *Cib2*^*KO*^ mice (Fig. 5e and Supplementary Fig. 5b, right panel). Further, immunoblotting revealed markedly reduced levels of mature-cathepsin D, consistent with the BODIPY-pepstatin A labeling, LAMP-1, and ATG5-ATG12 complex in *Cib2*^*KO/KO*^ mice, both one and twelve hours after light onset (Fig. 5f, g).

**Fig. 6:**
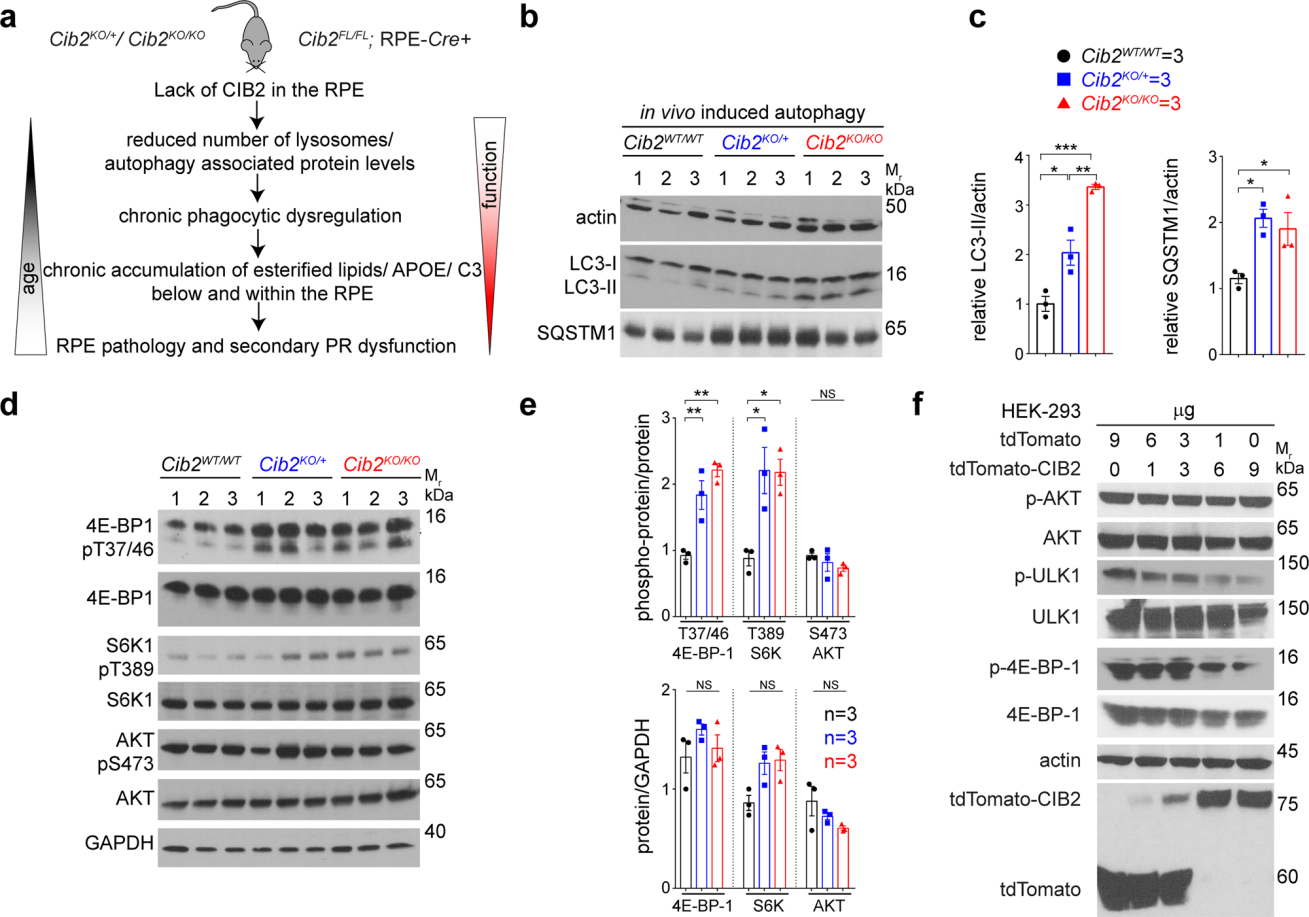
CIB2 modulates autophagy via the mTORC1 pathway. **a** Summary of eye pathophysiology found in *Cib2-*mutant mice. Global loss of single or both alleles of *Cib2* (*Cib2*^*KO/+*^, *Cib2*^*KO/KO*^), or specifically within the RPE (*Cib2*^*FL/FL*^; RPE-*Cre+*) leads to reduced non-canonical autophagy and chronic phagolysosomal processing defects, which gets exacerbated with age, leading to lipid and canonical AMD drusen markers accumulation below the RPE and secondarily leads to age-associated loss of photoreceptor (PR) function. **b**, **c** Induced autophagy in vivo by food starving for 24 h shows dysregulated autophagy in RPE/choroid lysates, as levels of LC3-II and p62/SQSTM1 are higher, quantified in **c**, in *Cib2*-mutant mice. **d**, **e** Higher phospho-protein levels, quantified in **e**, of mTORC1 targets 4E-BP-1 and S6K1, but not mTORC2 target AKT2, indicate hyperactive mTORC1 in RPE/choroid lysates after in vivo-induced autophagy. **f** Representative immunoblots of indicated phospho-proteins and proteins from HEK293 lysates over-expressing increasing amounts of tdTomato-CIB2 and decreasing amounts of tdTomato (total amount of plasmid DNA for each condition is 9 µg) causing reduction in mTORC1 activity. Data presented as mean ± SEM; each point represents an individual animal. One-way ANOVA and Bonferroni post hoc test (**c**, **e**). *p* < 0.05 (*), <0.01 (**), or <0.001 (***), NS not significant.

Complementary to the findings of *Cib2*-mutant mice, over-expressing CIB2 in RPE-J cells increased LAMP-1 and ATG5-ATG12 complex proteins levels (Supplementary Fig. 5c, d). To assess whether CIB2 overexpression has a functional impact, we performed in vitro pulse-chase phagocytosis assays^3^ followed by immunoblotting for opsin in RPE-J cells (schematic—Supplementary Fig. 5e, left panel). Again, in agreement with our findings in mice, we detected similar opsin levels bound initially (pulse-0 h) with control or CIB2 overexpression. However, 6 h later, corresponding to the digestion phase, we found less opsin in cells with excessive CIB2, suggesting that CIB2 overexpression alone suffices to boost phagolysosomal digestion (Supplementary Fig. 5e, f). Collectively, our results suggest that lack of CIB2, specifically in the RPE, causes defects in LC3-associated phagocytosis and reduced levels of autophagy proteins such as ATG5-12, in the RPE (Fig. 6a), whereas transient CIB2 overexpression augments OS clearance.

### Loss of CIB2 leads to autophagic clearance defects due to hyperactive mTORC1 signaling

Non-canonical autophagy such as phagolysosomal OS processing shares many aspects and proteins with canonical autophagy^2^. Hence, we reasoned that autophagy is impaired in *Cib2*-mutant mice. When autophagy is fully functional, the levels of autophagosome membrane protein LC3-II increase, while those of the autophagosome marker protein p62/SQSTM1 decrease in concert, due to specific digestion within the autolysosome. When autophagy is faulty, levels of both p62/SQSTM1 and LC3-II increase^34^. Thus, to assess the functionality, we evaluated the levels of p62/SQSTM1 and LC3-II in *Cib2*-mutant mice after inducing autophagy.

First, to induce autophagy in vivo, we fasted 2–3-months-old animals for 24 h and collected the RPE/choroid. Immunoblots of RPE/choroid lysates from autophagy induced *Cib2*^*KO/+*^ and *Cib2*^*KO/KO*^ mice showed that their p62/SQSTM1 and LC3-II levels doubled compared to those in WT age-matched controls (Fig. 6b, c). To assess if autophagy is dysregulated over time, we assessed the LC3-II and p62/SQSTM1 levels in aged mice, fed ad libitum. We observed a 2–3-fold increase in both LC3-II and p62/SQSTM1 levels, which suggests that loss of CIB2 leads to early as well as age-related autophagy deficits (supplementary Fig. 6a, b). LC3-I is a cytosolic protein but when lipidated with a phosphatidylethanolamine adduct (LC3-II form) localizes to the growing auto/phagosome membrane, and thus can be used as a reliable readout for the dynamic autophagic process^2,4,34^. RPE/choroid whole mounts, ex vivo, were incubated overnight with DMSO (control) or bafilomycin A1, which blocks autophagosome fusion with lysosomes, and the relative ratio of LC3-II (bafilomycin A1/DMSO) was assessed with immunoblotting^34^. We found a ~50% reduction in relative LC3 flux in RPE-specific *Cib2*^*KO*^ mice (Supplementary Fig. 6c, d). In addition, over-expressing CIB2 in RPE-J cells markedly increased relative LC3 flux in the presence of bafilomycin A1. The mTORC1 inhibitor rapamycin similarly elevated LC3-II levels (Supplementary Fig. 6e, f). These results strongly support the role of CIB2 in the clearance of autophagosomes, without compromising autophagosome biogenesis.

Since mTORC1 is a known negative regulator of autophagy, we assessed the status of mTORC1 signaling in *Cib2*-mutant mice. First, we measured phospho-protein levels of the bonafide mTORC1 downstream targets 4E binding protein 1 (4E-BP1) and S6 kinase 1 (S6K1). RPE/choroid lysate immunoblots from mutant mice undergoing induced autophagy revealed an approximately 2-fold increase in 4E-BP1 and S6K1 phospho-protein levels, but we found no difference in levels of the mTORC2 downstream target phospho-AKT. (Fig. 6d, e). Our kinase assay using mice brain lysates, further confirm mTORC1 hyperactivity in *Cib2*-mutant mice (supplementary Fig. 6g). Next, we tested if CIB2 is a specific negative regulator of mTORC1, mTORC2, or both. For this purpose, we used HEK293 cells for two reasons: first, because the mTORC1 signaling pathway is well-established in these cells^35^, and secondly to expand CIB2 functions beyond the RPE. HEK293 cells over-expressing increasing amounts of CIB2 showed decreasing phosphorylation of the mTORC1 targets 4E-BP1, and ULK1. We found no changes in mTORC2-mediated phosphorylation of AKT (Fig. 6f). Together, these results suggest that CIB2 impacts autophagy, specifically by modulating mTORC1.

### CIB2 forms a tripartite complex with Raptor-mTOR

**Fig. 7:**
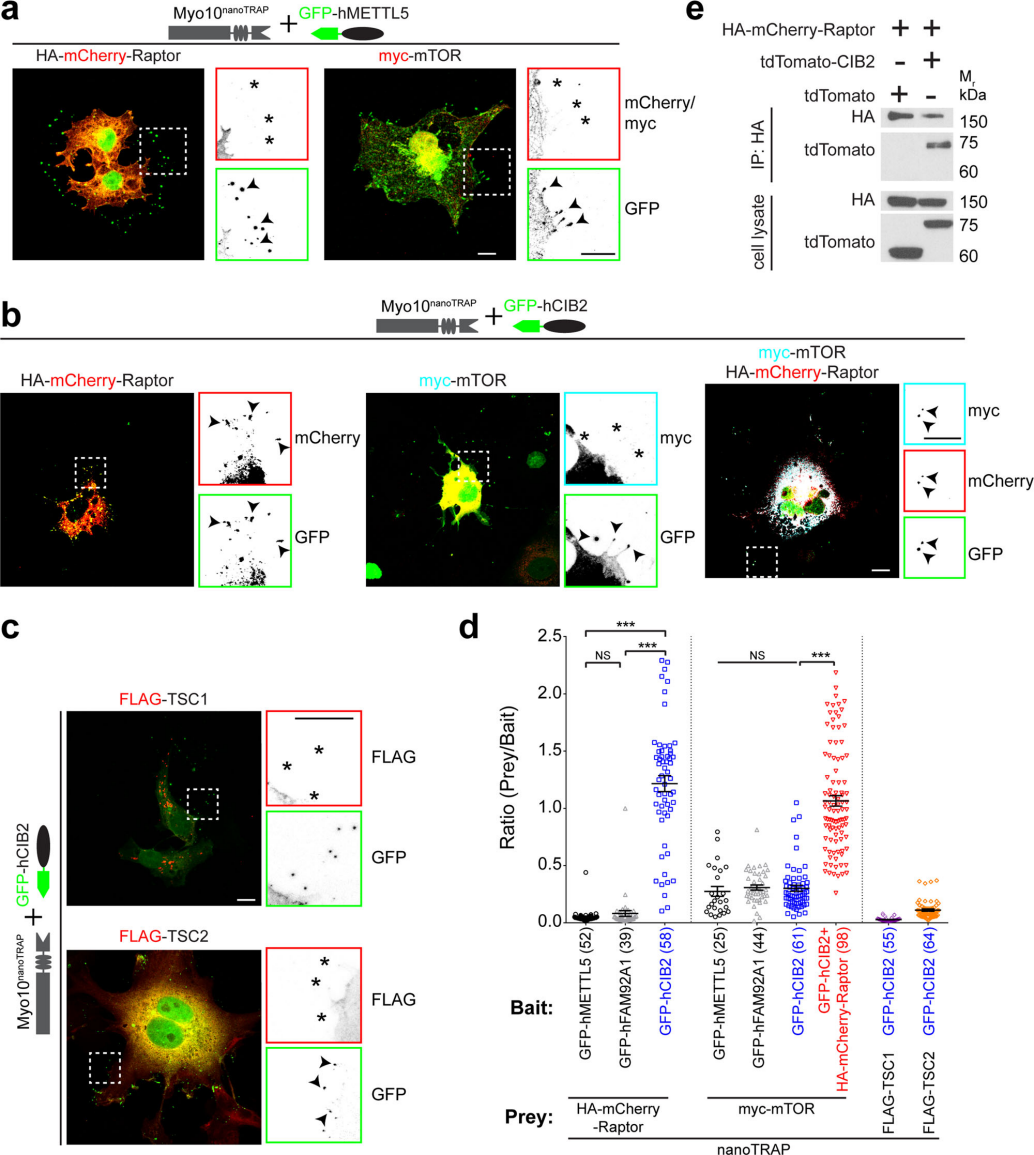
CIB2 interacts with Raptor and forms a tripartite complex with mTOR via Raptor. **a** Merged confocal micrographs of the HA-mCherry-Raptor (left panel) and myc-mTOR (right panel) constructs transiently transfected with nanoTRAP and GFP-hMETTL5 (controls) in COS-7 cells. Zoomed images of the inset show reverse color images of indicated constructs at the tip of filopodia. Arrowheads indicate accumulation of GFP-tagged protein at the tip of filopodia, * indicates the absence of the indicated construct in the red channel at the filopodia tips, suggesting no interaction of tagged proteins with the nanoTRAP and control GFP-METTL5 construct. Similar results were obtained with nanoTRAP and GFP-FAM92A1 (control) construct. **b** Merged images for indicated constructs in green: GFP-hCIB2, cyan: myc-mTOR, red: HA-mCherry-Raptor indicating that mTOR does not interact directly with CIB2 but forms a tripartite complex with Raptor and CIB2. Zoomed images of the inset show reverse color images of indicated constructs at the tip of filopodia. **c** Confocal images for FLAG-tagged TSC1 (top panel) and TSC2 (bottom panel) and nanoTRAP plus GFP-hCIB2 show no interaction for either protein with GFP-hCIB2. **d** Quantification for nanoTRAP assay for images shown in **a**–**c**. The number of filopodia tips analyzed is indicated in brackets. At least 3–5 cells for each condition from at least two independent experiments were analyzed. Data presented as mean ± SEM; each point represents an individual filopodia tip fluorescence ratio. One-way ANOVA and Bonferroni post hoc test, *p* < 0.001 (***), NS not significant. **e** Co-IP experiments in HEK293 cells show the interaction between exogenously expressed Raptor and CIB2. Scale bar: 10 µm.

To gain insights into the molecular mechanism of CIB2-mediated mTORC1 modulation, we assessed direct interactions between CIB2 and TSC1, TSC2, mTORC1-specific subunit Raptor, mTOR kinase, and Rheb, using a previously reported nanoscale pulldown 2.0 (NanoSPD) quantitative interaction assay^36^. NanoSPD utilizes a construct (nanoTRAP) consisting of GFP nanobody fused with the heavy meromyosin (HMM domain) of myosin 10 (myo10^HMM^-GFP nanobody). This allows preferential migration of any GFP-tagged protein to the filopodia tips in the transfected cells^36^. As anticipated, GFP-hCIB2 (bait) migrated efficiently to filopodia in COS-7 cells only in the presence of the nanoTRAP. As control, we ruled out the interaction of HA-mCherry-Raptor or myc-mTOR with nanoTRAP and with GFP tag (Fig. 7a, d). Using NanoSPD assay, TSC1 and TSC2, the major proteins of the TSC complex directly regulating Rheb, did not show any co-accumulation with GFP-hCIB2 (Fig. 7c, d). In contrast, we found that Raptor, but not mTOR, interacts with CIB2. However, mTOR and CIB2 can form a tripartite complex in the presence of Raptor (Fig. 7b, d). We further confirmed the CIB2-Raptor interaction using co-immunoprecipitation (co-IP) assay (Fig. 7e).

mTORC1 is a cytosolic complex when starved of amino acids and nutrients. However, it localizes to the lysosomes in the presence of amino acid, nutrients, and growth factors^12,13^. We sought to assess whether the loss of CIB2 leads to aberrant localization of the mTORC1 complex. First, we generated adipose tissue-derived mesenchymal stem cells (MSCs) from WT and *Cib2*^*KO/KO*^ mice (supplementary Fig. 7) to monitor the localization of the mTORC1 complex. In both WT and *Cib2*^*KO/KO*^ MSCs mTOR was cytosolic in the absence of amino acids (Supplementary Fig. 8a). Next, we triggered lysosomal localization by adding amino acids in the media. In both WT and *Cib2*^*KO/KO*^ MSCs, mTOR was co-localized with lysosomal marker LAMP, indicating that the loss of CIB2 did not affect the lysosomal targeting of mTOR trigged by amino acids (Supplementary Fig. 8a). Similarly, we observed no localization deficits in the absence and presence of other signaling factors such as insulin and glucose (Supplementary Fig. 8b, c). However, in the absence of CIB2, mTOR co-localized with LAMP-1 to a much greater extent, when Ca^++^ was chelated with EGTA (Supplementary Fig. 8d). In WT MSCs, we also assessed the co-location of CIB2 with TSC2. We found no significant co-localization between CIB2 and TSC2 under any of the conditions tested (supplementary Fig. 9a). These findings imply that CIB2 has a role in the Ca^++^-dependent regulation of mTORC1 complex localization in the lysosomes.

### CIB2 negatively regulates mTORC1 via preferential binding to “nucleotide empty-” or GDP-Rheb

**Fig. 8:**
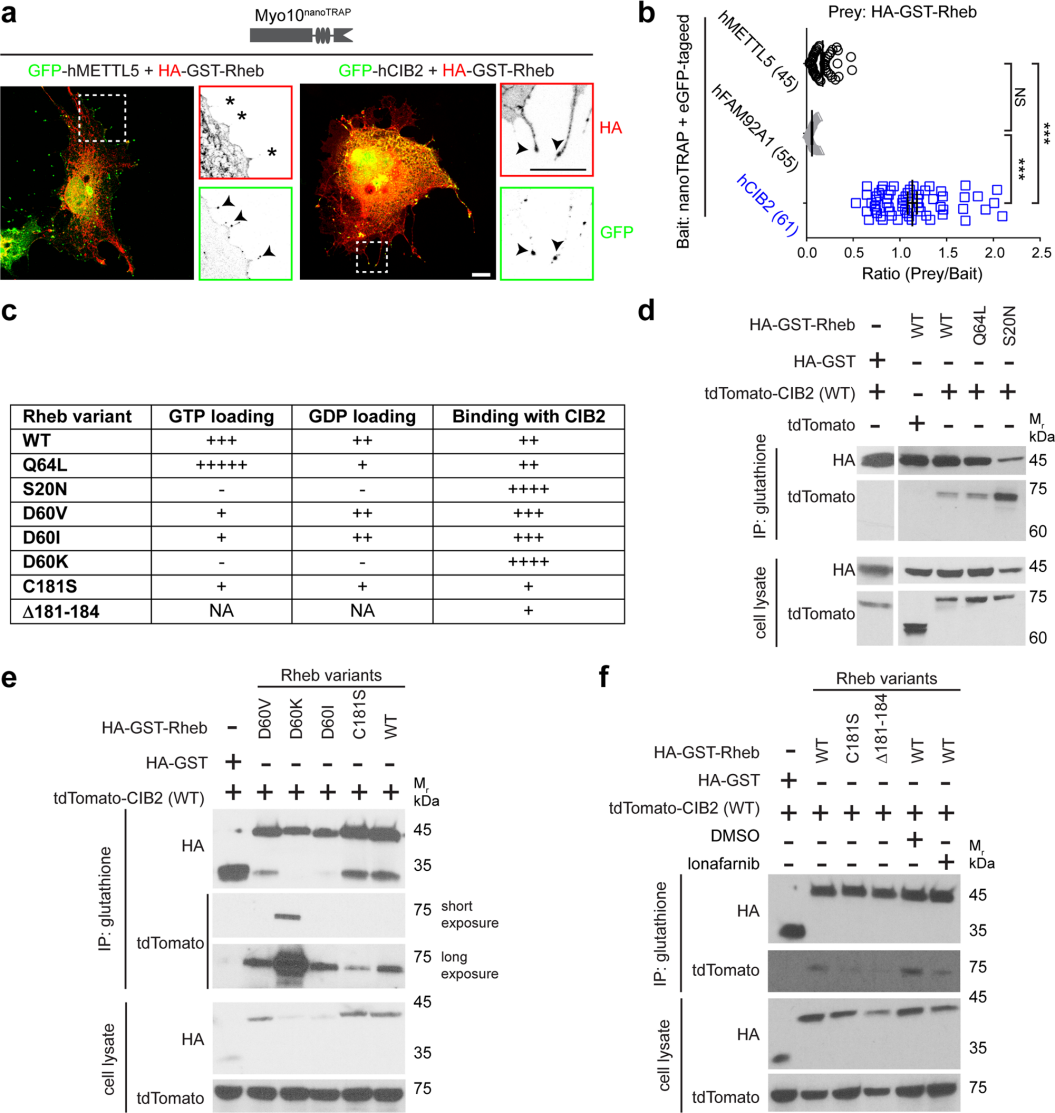
CIB2 modulates mTORC1 signaling via preferential binding to Rheb-GDP. **a**, **b** Merged representative confocal micrographs of bait GFP-hMETTL5 (green, left panel, control) or GFP-hCIB2 (right panel) with prey HA-GST-Rheb (red), and nanoTRAP in COS-7 cells. Zoomed images of the inset show reverse color images of indicated constructs at the tip of filopodia. * indicates absence at the filopodia tips demonstrating no interaction of HA-GST-Rheb with the GFP-h METTL5. Arrowheads indicate accumulation of indicated construct at the tip of filopodia. The right panel shows the interaction of CIB2 with Rheb, the mTORC1 activating GTPase. Scale bar: 10 µm. The prey/bait ratio is quantified in **b**. The number of filopodia tips analyzed is indicated in brackets. At least 3–5 cells for each condition from at least two independent experiments were analyzed. Data presented as mean ± SEM; each point represents an individual filopodia tip fluorescence ratio. One-way ANOVA and Bonferroni post hoc test, *p* < 0.001 (***), NS not significant. **c** Strength of CIB2 binding with various Rheb variants from **d**–**f**, and the relative GTP and GDP binding of Rheb variants is estimated from previous studies^38–40^. NA data not available. **d** Representative immunoblots of co-IP experiments with indicated HA-GST-Rheb constructs with WT tdTomato-CIB2 show stronger binding with the “nucleotide empty” Rheb-S20N variant. **e** Representative immunoblots of co-IP experiments with indicated HA-GST-Rheb constructs with WT tdTomato-CIB2 show stronger binding with the “nucleotide empty” Rheb-D60K variant and reduced binding with the farnesyl mutant Rheb-C181S. Short exposure ~1 min, long exposure ~10 min. **f** Representative immunoblots of co-IP experiments with indicated HA-GST-Rheb constructs with WT tdTomato-CIB2 show reduced binding with the farnesyl mutants Rheb-C181S and Δ181–184, and cells treated with farnesyl transferase inhibitor lonafarnib (1000 nM).

When we analyzed the co-localization of CIB2 with Rheb, we found significant overlap under all the conditions tested (supplementary Fig. 9b). To assess whether CIB2 actually directly interacts with Rheb we utilized the NanoSPD assay. Interestingly, NanoSPD revealed a direct interaction between CIB2 and Rheb (Fig. 8a, b), adding a new binding partner into the growing list of Rheb interactome^37^. CIB2 partnered uniquely with Rheb, but not with other Rheb-activators; including TSC complex and microspherule protein 1 (Supplementary Fig. 10a–c). Rheb is a 184 amino acid evolutionary conserved GTPase (Supplementary Fig. 11). GTP-bound Rheb acts as a potent and obligate activator of mTORC1^15^. Hence, we next explored the impact of the nucleotide-binding state of Rheb on interaction with CIB2 through co-IP studies (Fig. 8c–f). For these studies, we used WT protein, Rheb harboring the Q64L variant that has increased GTP loading and partial resistance to GAP activity of TSC, and Rheb with S20N missense variant that has severely reduced GTP and GDP binding capacity (~1% of WT Rheb), essentially acting as a “nucleotide empty” variant^38^. Consistent with prior findings, we found reduced expression of Rheb-S20N variant protein (Fig. 8d – cell lysate, Supplementary Fig. 11d). In the co-IP assay, CIB2 bound preferentially to the S20N variant as compared to either the Rheb-WT or the Rheb-Q64L variant (Fig. 8d).

CIB2’s preferential binding to nucleotide reduced/empty state of Rheb was further validated by loading glutathione bead-immobilized HA-GST-Rheb (WT) with either GDP or non-cleavable GTPγS and adding CIB2 lysate (Supplementary Fig. 11b), and through analyzing additional mutagenic constructs (nucleotide-binding capacity summarized in Fig. 8c). Previously, a mutagenesis screen and subsequent targeted substitution of aspartic acid at position 60 (D60) to valine (V) or isoleucine (I) showed significantly diminished affinity to GTP but comparable GDP binding capacity as WT Rheb. Meanwhile, the D60K variant turned out to be a nucleotide empty variant^39,40^. In our co-IP studies, we found that CIB2 preferentially binds to, in order of binding strength, nucleotide empty D60K variant, than D60I and D60V (Fig. 8e).

Farnesylation of Rheb is essential for nucleotides loading and activation^38^. To explore the essentiality of Rheb-farnesylation for CIB2 coupling, we performed co-IP studies using the farnesylation deficient variant C181S, and Rheb with the last four amino acids deleted (Δ181–184), which was shown to be essential for proper loading of nucleotides^38^. We found significantly reduced CIB2 binding in the absence of Rheb-farnesylation (Fig. 8e, f). These findings were further confirmed by using the farnesyl transferase inhibitor, lonafarnib (Fig. 8f). Finally, as we observed CIB2 interaction both with Rheb and Raptor, which are also known to interact with each other^41^, we wondered if CIB2 impacts the interaction of Rheb with Raptor. However, no obvious effect on the binding of Rheb to Raptor in the presence of CIB2 was observed in co-IP assays, which suggests non-competing binding sites of Raptor and CIB2 with Rheb (Supplementary Fig. 11c). Taken together, our studies demonstrated that CIB2 modulates mTORC1 activity, and hence autophagy, by preferentially binding to nucleotide empty/GDP-Rheb (inactive form).

### mTORC1 is also upregulated in RPE/choroid tissues from patients affected with AMD

**Fig. 9:**
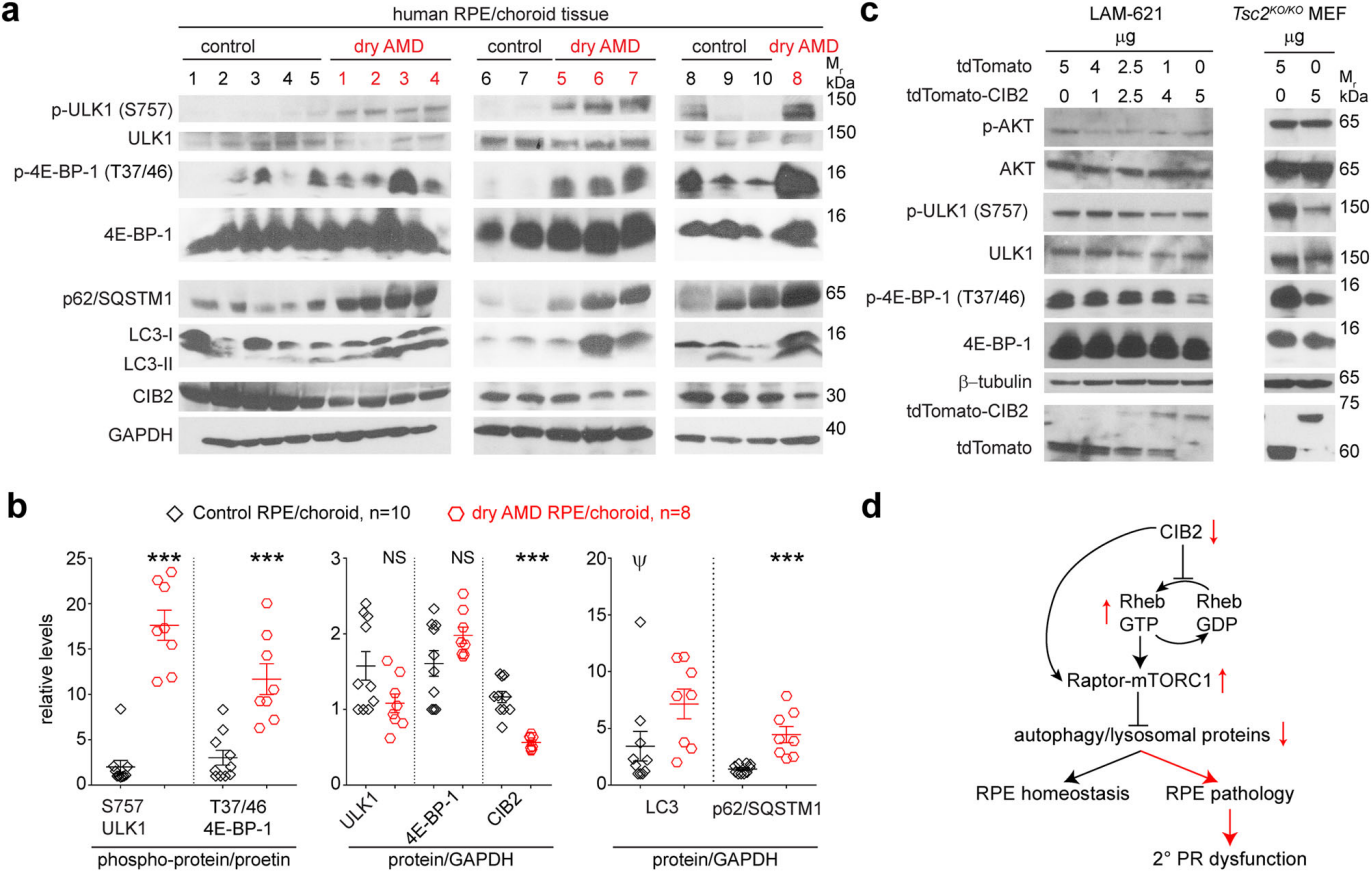
mTORC1 signaling and CIB2 levels are dysregulated in RPE/choroid lysates from dry AMD cases, while over-expressing CIB2 downregulates mTORC1 signaling in cell lines. **a**, **b** Immunoblots from RPE/choroid lysates from dry AMD (*n* = 8) or control (*n* = 10) age-matched donors shows reduced levels of CIB2, accumulation of autophagy proteins, and over-active mTORC1 signaling, quantified in **b**, concordant to the molecular deficits found in RPE of *Cib2-*mutant mice. Data presented as mean ± SEM; each data point represents an individual donor. Unpaired two-tailed *t*-test, *p* < 0.05 (*), <0.01 (**), and <0.001 (***). NS not significant. Please note that one control sample (*ψ*) is an outliner and is highly variant from the rest. Re-quantification, omitting the highly variant control sample, is shown in Supplementary Fig. 12. **c** Immunoblots from lymphangioleiomyomatosis patient-derived cell line (LAM-621; left panel) and *Tsc2*^*KO/KO*^, *p53*^−^^*/−*^ mouse embryonic fibroblasts (MEF; right panel) lysates over-expressing either tdTomato or CIB2. Intriguingly, CIB2 can partially reduce the over-active mTORC1 signaling even in the absence of TSC. **d** Model of CIB2 function in retinal sensory cells. In our model, CIB2 is critical for regulating mTORC1 signaling and autophagy in RPE via its preferential interaction with nucleotide empty or GDP-Rheb (inactive form) and raptor. Loss of CIB2 results in reduced autophagy and exacerbated mTORC1 signaling, thus leading to RPE pathology and secondary photoreceptor (PR) dysfunction.

Previous studies in mouse models^2–4,42,43^ and humans samples^5,42,44^ have shown that both non-canonical and canonical autophagy is essential for RPE function. In humans, AMD is characterized by drusen accumulation in the inner collagenous layer of Bruch’s membrane, RPE vacuolization, and accumulation of lipid deposits within the RPE, Bruch’s membrane, and elsewhere. Aging-associated lipid accumulation is further thought to hinder lipid degradation by phagolysosomes and autolysosomes in RPE, thereby exacerbating the accumulation of undigested lipids^45^, and this has been suggested as an important causative factor in dry AMD. Since our CIB2-deficient mice showed similar age-related RPE pathologies, we reasoned that upregulated mTORC1 signaling might be a contributing factor for the autophagy deficits seen in AMD patients as well. To test this, we evaluated RPE/choroid tissues bio-banked from human subjects affected with dry AMD (*n* = 8) along with age-matched control (*n* = 10) tissues (Supplemental Table 1). Consistent with observations made in primary RPE cell cultured from AMD patients^5^, autophagy was impaired in dry AMD RPE/choroid lysates, as exhibited by the accumulation of both p62/SQSTM1 and LC3. Further, dry AMD patients’ lysates exhibited reduced CIB2 expression, and higher levels of phosphorylated, but not of total protein levels, ULK1 and 4E-BP1 (Fig. 9a, b and Supplementary Fig. 12), indicating hyperactive mTORC1 signaling. These results demonstrate that similar to *Cib2-*mutant mice, dry AMD patients’ RPE/choroid tissue also has reduced CIB2 levels, hyperactive mTORC1 signaling accompanied by autophagy deficits.

Besides AMD (shown here), upregulated mTORC1 signaling is also implicated in aging, TSC, and cancers, including lymphangioleiomyomatosis (LAM). Further, TSC variants lead to RPE lesions in human patients^46^. The above studies demonstrated that CIB2 acts as a negative regulator of mTORC1, and hence, we reasoned that CIB2 overexpression may partially downregulate the hyperactivated mTORC1 signaling observed in TSC and LAM. To evaluate this, we utilized two different diseases models. First, a patient-derived cell line LAM-621 (LAM-associated renal angiomyolipoma) harboring a variant in TSC2 (p.Arg611Gln) rendering it unable to complex with TSC1, resulting in hyperactivated mTORC1 signaling^47^. LAM-621 cells over-expressing increasing amounts of CIB2, showed decreasing amounts of phosphorylated 4E-BP-1 and ULK1, but not AKT (Fig. 9c, left panel). Secondly, *Tsc2*^*KO/KO*^, *p53*^*KO/KO*^ mouse embryonic fibroblasts (MEFs), similarly over-expressing CIB2, resulted in a reduction of mTORC1 but not mTORC2 targets (Fig. 9c, right panel). Taken together, we show that CIB2 preferentially binds to an inactive form of Rheb, thereby regulating mTORC1 activity. Aberrant mTORC1 activity leads to chronic autophagy deficits leading to RPE pathology and secondary photoreceptor dysfunction (Fig. 9d). Our findings signify that CIB2 can partially downregulate mTORC1, independently of the TSC complex, and thus potentiating it as a target for modulation of mTORC1 specifically.

### Rescue of PR function in *Cib2*-mutant mice by evading the RPE- retinoid cycle

**Fig. 10:**
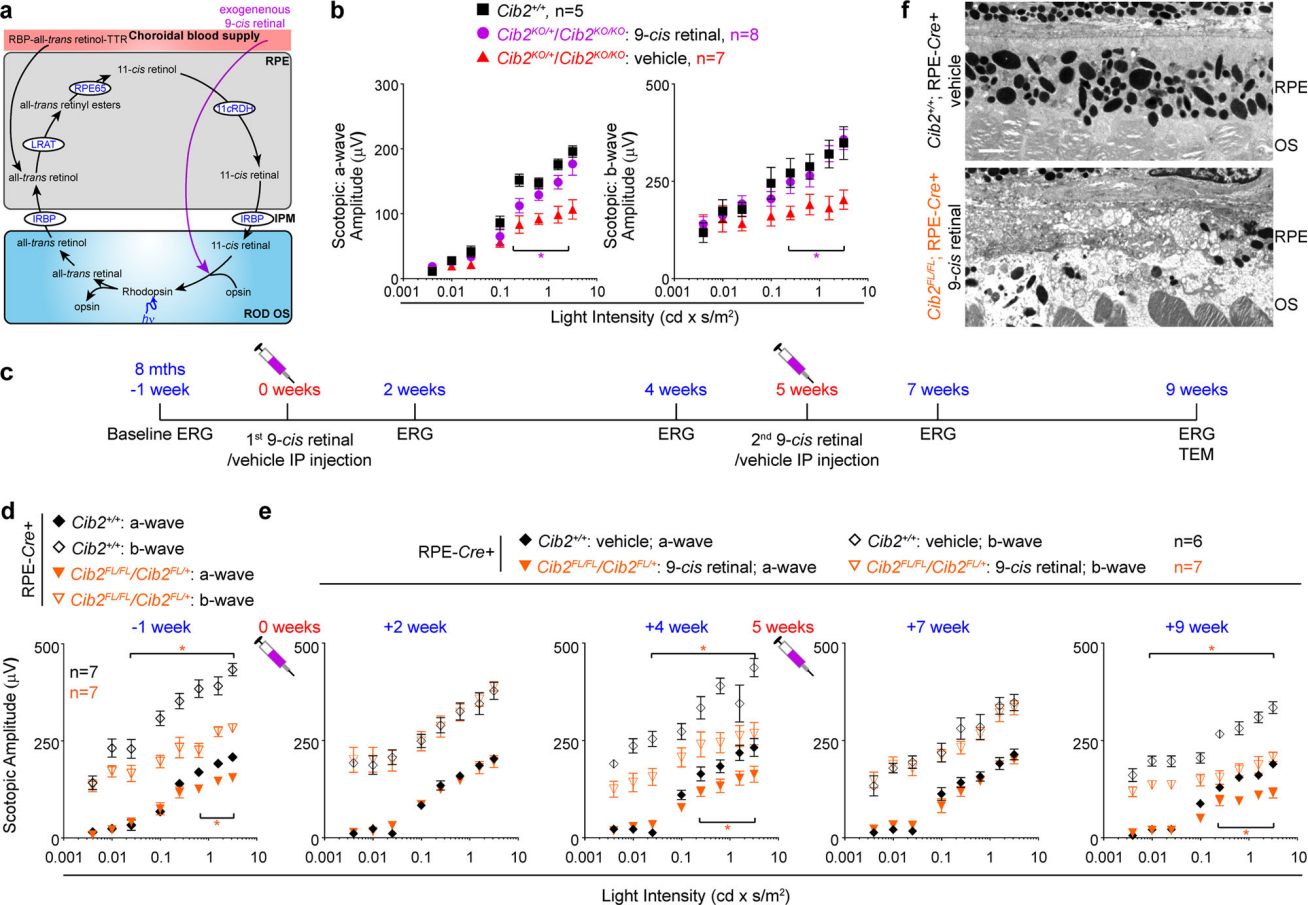
PR function but not RPE pathology can be rescued by exogenous retinoid. **a** Schematic of the visual cycle and exogenous 9-*cis* retinal treatment. **b** Eight-to-nine-months-old *Cib2*-mutant mice (*Cib2*^*KO/+*^ and *Cib2*^*KO/KO*^) were either intraperitoneally injected with vehicle (*n* = 7) or 0.25 mg 9-*cis* retinal (*n* = 8), dark-adapted overnight, and ERG performed the next day. Both a-wave (left panel) and b-wave (right panel) amplitudes were higher in mutant mice injected with 9-*cis* retinal than vehicle-injected mice. For comparison, untreated WT control mice (black squares, *n* = 5) are replotted from Fig. 1c, bottom panels. **c** Schematic of treatment paradigm in RPE-specific *Cib2* mutant mice injected with 9-*cis* retinal and control mice injected with vehicle. **d** Baseline ERG for indicated genotype is reproduced from Fig. 2a, bottom panels. **e** ERG amplitudes for indicated genotype after specific treatment as outlined in **c** (vehicle-injected; *n* = 6, 9-*cis* retinal injected *n* = 7). **f** TEM micrographs for indicated genotype at the end of the treatment paradigm as outlined in **c** (*n* = 3/genotype). Scale bar, 2 µm. Data presented as mean ± SEM; each data point represents an individual animal. Unpaired two-tailed *t-*test, *p* < 0.05 (*).

RPE is essential for the visual cycle regeneration of 11-*cis* retinal, the chromophore of photoreceptor opsins. Upon photon absorption, 11-*cis* retinal is photo-isomerized to all-*trans* retinal, thereby activating opsin and initiating the phototransduction cascade^48,49^. All-*trans* retinal is regenerated to 11-*cis* retinal, which is then recycled back to the photoreceptors via a series of transport and enzymatic steps, occurring primarily in the RPE (illustrated in Fig. 10a). The ERG deficits in *Cib2* mutants without significant photoreceptor damage led us to investigate this key visual pathway. First, we quantified the absolute retinoid levels (11-*cis* oxime, all-*trans* oxime, retinyl esters, and A2E) within the retina or RPE/choroid in 2-to-3 months and 8-to-9-months-old *Cib2*^*KO/+*^, *Cib2*^*KO/KO*^. However, no significant differences were observed when compared to control age-matched WT mice (Supplementary Fig. 13a, b). We reasoned that the vacuoles observed in the RPE may partially sequester retinoids, rendering them unavailable to re-enter the PR. Therefore, we tested whether we could rescue the ERG defects via exogenous chromophore, thus bypassing the RPE visual cycle altogether. For these studies, we used two different treatment paradigms. First, we injected aged global *Cib2*^*KO*^ (either heterozygous or homozygous) mice once with either 9-*cis* retinal (an analog of 11-*cis* retinal) or vehicle. Then, we assessed their visual function by ERG the following day, after dark adaptation. Treatment of *Cib2*^*KO*^ mice with 9-*cis* retinal restored their ERG amplitudes to near WT levels with significant differences in scotopic a- and b-wave amplitudes as compared to vehicle-injected *Cib2*^*KO*^ controls (Fig. 10b).

Second, to further assess the potential clinical translatability of exogenous retinoids, we employed an extended treatment paradigm following injection of 9-*cis* retinal in RPE-specific *Cib2*^*KO*^ mice (Fig. 10c). We performed baseline ERGs on 8-months-old uninjected mice and found significant differences in ERG amplitudes in mutant mice compared to RPE-*Cre* + control mice (Fig. 10d). Two weeks after the first injection, we found significantly improved ERG amplitudes for RPE-specific mutant mice injected with 9-*cis* retinal. The ERG amplitudes of 9-*cis* retinal injected mice were essentially indistinguishable from those of vehicle-injected control mice. However, the rescue effect of exogenous retinoid was short-lived. By four weeks post-injection, the ERG amplitudes of 9-*cis* retinal injected RPE-specific mutant mice were substantially lower than control mice. To assess the medium-term efficacy of exogenous retinoid therapy, we extended the paradigm further by 1 month. Again, we saw an improvement in ERG amplitudes for mutant mice injected with 9-*cis* retinal two weeks after the second injection, but by four weeks, ERG amplitudes fell back to baseline levels (Fig. 10e). This suggested that short-term but reproducible improvement in ERG amplitudes is possible with this treatment. At the conclusion of treatment, TEM analysis of the RPE revealed vacuoles and/or sub-RPE deposits present in all three 9-*cis* retinal-treated RPE-specific *Cib2*^*KO*^ mice, but not in vehicle-treated RPE-*Cre* + control mice (1 of 3) (Fig. 10f). The results strongly suggest that repeated exogenous retinoid treatment can rescue photoreceptor function temporarily yet reproducibly in *Cib2*-deficient mice by bypassing the RPE. Although, (as expected) this does not improve RPE pathophysiology.

## Discussion

This study highlights the crucial role of CIB2 in the regulation of phagolysosomal digestion of OS and the development of age-related phenotype in mice. CIB2 belongs to a family of proteins that includes four members (CIB1–CIB4). CIB proteins contain Ca^2+^/Mg^2+^ binding EF-hand domains, and binding of these ions leads to a conformational change and downstream effects, similar to the well-studied protein, calmodulin^50,51^. In recent years, the various developmental or functional roles of CIB2 have been elucidated in multiple organs, however, the precise tissue-specific mechanisms remain unclear. For instance, *CIB2* is downregulated in ovarian cancer and associated with poor prognosis in ovarian cancer patients^52^. Previously, we and others reported on the essential function of CIB2 in the inner ear development and mechanotransduction of sound signal^18,53,54^. Furthermore, CIB2-deficient mice showed a small, but not statistically significant difference in ERG amplitude between heterozygous and homozygous mice, leading the authors to conclude that CIB2-deficient mice exhibit no retinal phenotype^53^. However, the main caveats of that study were use of only one intensity of light for retinal functional evaluation and the lack of instrumental WT controls^53^.

In this study, we used three different mutant mouse strains and found that both a complete loss (homozygous) and haploinsufficiency (heterozygous) of CIB2, specifically in RPE, leads to attenuated ERG amplitudes and development of age-related phenotype encapsulating several dry AMD features. The mutant mice from our study had fewer lysosomes, lysosomal and autophagy proteins, and defects in phagolysosomal clearance of OS. As OS phagosomes contain ~50% lipid, chronically delayed OS clearance causes accumulation of lipids. Confirming this trend, we found double the amount of neutral lipid droplets in the RPE in aged *Cib2*^*KO*^ mice with a gradual accumulation of un-cleared lipids and proteins. Since RPE cells are post-mitotic these insults accumulate over time, resulting in an age-dependent RPE pathology and PR dysfunction. Earlier, genetic deletion of genes involved in clearance of ingested OS, specifically in the RPE, was found to lead to secondary photoreceptor functional defects^2–4^. Further, the RPE presents the largest source of electrical resistance for in vivo ERGs^55^, and hence the ERG changes we observed were likely caused by RPE dysfunction and subsequent pathology. Our PR- and RPE-specific *Cib2* knockout data further support this conclusion.

The RPE-mediated OS non-inflammatory clearance process requires LC3-associated phagocytosis. Deficiency in LAP has been associated with various disorders such as SLE, multiple sclerosis, atherosclerosis, and AMD, amongst others^56^. It has proven quite challenging to study AMD, since mouse models of genetically validated targets do not recapitulate the human phenotype. Here, we show that global or RPE-specific lack of CIB2 recapitulates many aspects of the human AMD pathophysiology including sub-RPE deposits, and accumulation of APOE, C3, and aβ. Further, we found increased lipid droplets and esterified cholesterol, which is associated with drusen in humans and sub-RPE deposits in mouse models. Autophagy decreases with aging in several tissues including the RPE^9,42,44^. RPE cells cultured from AMD patients showed elevated numbers of autophagosomes, lipid droplets, aberrantly large lysosomes, and higher starvation-induced SQSTM1/p62 levels^5^. Consistent with these observations, we found an accumulation of both p62/SQSTM1 and LC3, suggesting aberrant autophagy in RPE/choroid samples from dry AMD patients. Concordant with our animal models, we also observed reduced levels of CIB2, and hyperactive mTORC1 in these human samples. Together, these data reconcile CIB2 levels, mTORC1 activity, autophagy, and their age-associated impact on the RPE maintenance and function in mice and humans.

mTORC1, the key negative regulator of autophagy, is cytosolic and in the presence of sufficient nutrients, it is recruited to the surface of the lysosomes^12,13^. GTP-loaded active Rheb allosterically realigns the kinase active site of mTORC1, and thus is a potent and obligate activator of mTORC1 on the surface of the lysosomes^15^. We found that CIB2 preferentially binds to, in order of binding strength, the nucleotide-empty form of Rheb followed by GDP-loaded inactive Rheb, and then GTP-loaded active Rheb. Our results here support the function of CIB2 as a “co-factor” to preferentially maintain Rheb in an inactive state. However, further biochemical studies will be needed to decipher the precise mechanism and stoichiometry of CIB2-Rheb and CIB2-Raptor interactions. Furthermore, since mTORC1 is downstream of AMP-activated protein kinase (AMPK), one of the main integrators of energy cues and a negative regulator of mTORC1^57^, it would be interesting to assess whether CIB2 establishes any feedback mechanism between mTORC1 and AMPK pathways. Lastly, we also found differential regulation of mTOR localization upon Ca^++^ chelation in the CIB2-mutant mice (supplementary Fig. 8d), alluding to the potential role of CIB2 in intracellular Ca^++^ regulation, as was found for CIB1^50^. Our current study establishes CIB2 at the intersection of autophagy, mTORC1 signaling, and visual function, thus providing fundamental knowledge that would be instrumental in gaining further mechanistic insights.

Besides AMD, a growing literature points to defects in autophagy, lysosomal functions, and mTORC1 signaling in aging and diverse neurodegenerative disorders with complex genetic architecture such as Alzheimer’s, autism, epilepsy, cancers, obesity, and heart diseases. Thus, identifying molecular targets that can be used to modulate or restore autophagy may offer new avenues for the treatment of such disorders^58^. Direct mTOR kinase inhibitors or rapamycin (and its analogs) have shown only limited success in dry AMD patient trials^59,60^, as chronic exposure to either class of compounds also leads to mTORC2 disassembly and affects cell survival^61^. Since direct mTOR inhibition is not a viable long-term strategy, there is intense efforts to discover drugs modulating the mTORC1 pathway by targeting other players of the pathway such as Rheb^62^. *CIB2* is a small gene that can easily fit within the carrying capacity of AAV for in vivo delivery, supporting potential translatability via gene therapy. Moreover, autophagy is linked to lysosomal biogenesis via the transcription factor EB (TFEB) family, the lysosomal master transcription factor family (TFEB/ TEF3/ MiTF) via which mTORC1 controls lysosomal biogenesis^63^. The reduction of lysosomal proteins in RPE of *Cib2-*mutant mice could potentially be mediated via mTORC1 regulation of TFEB. If so, CIB2 overexpression might be dually beneficial, since it would reduce mTORC1 activity and thereby upregulate autophagy and lysosomal biogenesis - promising topics for further basic or translational studies.

Developing translational models to study autophagy deficits is imperative to enable the evaluation of rational treatments for AMD. Patients with dry AMD, in particular, have little to no treatment options, although the AREDS (Age-Related Eye Disease Study) nutritional supplementation formula, with carotenoids, vitamins, zinc, and copper, have been shown to have a modest effect on progression to neovascular AMD. Bypassing the RPE visual cycle by supplying exogenous retinoids has been found to effectively serve as a useful therapeutic approach for certain retinal disorders^64,65^. The most common methodology is using a 9-*cis* retinoid analog that is more stable than the physiological 11-*cis* retinal chromophore. Upon entering the PR, 9-*cis* retinal can bind with opsin to create functional visual pigment. However, unbound retinals are quite cytotoxic and quickly detoxified by the liver—an issue that has been circumvented by using the less toxic oral 9-*cis* retinyl acetate to treat inherited blindness of childhood caused by RPE65 and LRAT mutations^66^. We have found that *Cib2*^*KO*^ RPE contains abundant large lipid-filled vacuoles and sub-retinal deposits, which may impede RPE transport mechanisms including those that deliver retinoids back to the PR, leading to reduced ERG amplitudes, even without gross PR damage. Supporting this idea, we found that providing exogenous retinoids improved ERGs in two different treatment paradigms in two different mouse models, thus providing a preclinical proof-of-concept for use of retinoids to treat AMD.

## Methods

### Animals, tissue harvest, and processing

The ARRIVE guidelines for reporting animal research were used for procedures involving animals. All animal studies were conducted according to the ARVO Statement for the Use of Animals in Ophthalmic and Vision Research and the National Institutes of Health Guide for the Care and Use of Laboratory Animals, and after approval from the IACUC (Institutional Animal Care and Use Committees) of the University of Maryland School of Medicine. *Cib2*^*tm1a*^ mice (designated *CIB2*^*KO*^) were purchased from EUCOMM and have been validated previously^18,53^. To generate floxed allele (*Cib2*^*flox*^), *Cib2*^*tm1a*^ were crossed ROSA26::FLPe knock in mice (Jax lab, stock#003946), which resulted in the removal of LacZ-neomycin selection cassette by Flp-FRT recombination. RPE-specific *Cre*-expressing mice were described earlier^21^, and were generously shared with us by Drs. Sheldon Miller, National Eye Institute and Joshua Duniaef from University of Pennsylvania. Previous research pointed out that these mice may develop *Cre*-mediated age-dependent RPE pathology^67^, hence for all experiments we used *Cib2*^*+/+*^; RPE-*Cre+* mice as controls. Rod photoreceptor-specific *Cre*-expressing mice^20^ were purchased from Jackson Labs. Previous studies and our own studies have not shown any *Cre*-mediated toxicity in this line, hence for controls we used a mix of *Cib2*^*flox/flox*^ or *Cib2*^*flox/+*^*; Cre-*, and *Cib2*^*+/+*^; *Cre* + mice. Mice were housed under a strict 12:12 h light on/off cycle and fed standard mouse diet (after weaning) and water ad libitum. Mice were euthanized by CO_2_ asphyxiation followed by cervical dislocation before immediate eye enucleation. Eyes were immersion-fixed in 2% PFA + 2% glutaraldehyde for morphometry and TEM.

RPE flat mounts were generated by first removing the anterior segment and lens from an eye, and then removing the retina carefully in Hank’s balanced salt solution (HBSS). After fixing the eyecup in 4% PFA in PBS for 30 min to 1 h, radial cuts were made to flatten the eyecup, and antibody/dye labeling was performed for fluorescence microscopy. For immunoblots, RPE/choroid tissues were snap-frozen on dry ice before storing at −80 °C before lysis. For retinoid analysis, mice were dark-adapted overnight, and dissections were performed under dim red light. For autophagy flux assay, whole mounts of RPE/choroid tissues were incubated in DMSO (control) or 50 nM Bafilomycin A1 overnight in DMEM at 37 °C before rinsing with PBS, tap drying on filter paper, followed by snap-freezing on dry ice and storing at 80 °C. Finally, for assessing mTORC1 activity following increased autophagy in vivo, mice were fasted with unlimited access to water for 24 h, followed by RPE/choroid dissection as above.

### Morphometric analysis of the outer retina

Plastic sections were prepared as previously described^68^. Eyes were hemisected from the pupil through the optic nerve and the eyecups harvested, dehydrated in ascending ethanol concentrations, infiltrated, and embedded in JB-4 Plus resin (Ted Pella, Redding, CA). Sections 4 µm thick were cut on a Leica EMUC6 Ultramicrotome (Leica Microsystems, Buffalo Grove, IL), mounted on slides, dried, and stained with 1% cresyl violet (Sigma, St. Louis, MO). Sections were examined at ×40 or ×100 using a NIKON EFD-3 Episcopic-Fluorescence microscope (Nikon Inc., Melville, NY). Photomicrographs were taken using a Moticam 2500 high-resolution camera (Motic, British Columbia, Canada), and brightness and contrast adjusted as needed using Adobe Photoshop (Adobe Systems, San Jose, CA).

To measure the thicknesses of outer retina layers, a vertical line was drawn from the outer plexiform layer (OPL) to the RPE, and the OPL, outer nuclear layer (ONL), PR OS, and distances of inner segment (IS) edges measured individually from this line using Moticam Image Plus 2.0 Software (Motic China Group Co., Ltd., Xiamen, China), in 10 sections per eye, and overall means calculated. Retinal thickness was measured without knowledge of the genotype by a masked observer (PAS).

### Transmission electron microscopy

Sections for EM were prepared as previously described^68^. Briefly, hemisected eyecups were rinsed in buffer and postfixed in 2% osmium tetroxide and 1.5% potassium ferrocyanide in dH_2_O for 2 h, dehydrated in a graded ethanol series, and embedded in Epon-Araldite (Electron Microscopy Sciences, Hatfield, PA). Semi-thin sections (4 μm) were cut and stained with 1% cresyl violet. Ultra-thin sections (90 nm) were cut on an ultramicrotome (Ultracut E 701704, Reichert-Jung, Buffalo, NY) using a diamond knife (Micro Star Technologies, Inc., Huntsville, TX), collected on copper grids, counterstained with 4% methanolic uranyl acetate (Electron Microscopy Sciences, Hatfield, PA), and outer retinal morphology examined using a transmission electron microscope (TEM; Model 300: Phillips, Eindhoven, The Netherlands). Photomicrographs were captured with a digital camera (15-megapixel digital camera, Scientific Instruments and Applications, Duluth, GA) and Maxim DL Version 5 software (Diffraction Limited, Ottawa, Canada).

### Electroretinography

Electroretinograms (ERG) were recorded as earlier described^3^ with modifications. Mice, overnight dark-adapted, were anesthetized with ketamine-xylazine (100 and 10 mg/kg, respectively) and pupils were dilated with 1% Tropicamide. A gold wire lens electrode was placed on the cornea, a platinum reference electrode in the mouth, and a ground electrode on the tail. To assess rod-driven responses, increasing scotopic stimuli were presented sequentially (0.003962233 to 3.147314 cd × s/m^2^) at 5–60 s intervals using UTAS BigShot (LKC Technologies, Gaithersburg, MD). At least 3 waveforms per intensity were averaged. For cone function evaluation, photopic responses to a single bright flash (3.15 cd × s/m^2^) under a steady rod-suppressing field of cd × s/m^2^. Waves were analyzed using EM for Windows software (LKC Technologies). For 9-*cis* retinal treatment, animals received intraperitoneal 0.25 mg 9-*cis* retinal (dissolved in 100% Et-OH) in a 200 µl vehicle (10% BSA in 0.9% NaCl solution, sterile filtered) or vehicle only^69^, in the dark. ERGs were performed as above, the next day, or at indicated times.

### Cell culture, drug treatment, synchronized cell culture phagocytosis assay

Immortalized RPE-J cells derived from rat (ATCC, Manassas, VA) were maintained at 32 °C and 8% CO_2_ in 4% FBS/DMEM supplemented with 1x penicillin/streptomycin. HEK-293T, COS-7, LAM-621, and *TSC2*^*KO/KO*^ *p53*^*KO/KO*^ MEF cells were maintained at 37 °C/5% CO_2_ in 10% FBS-DMEM supplemented with 1× penicillin/streptomycin. OS were purified from fresh porcine eyes harvested within 24 h from Sierra For Medical Science (Whittier, CA) using an established protocol^70^, modifying only the preparation of sucrose density gradient tubes. Twenty-four hours before OS purification, sucrose step gradients (20–60% in 10% increments) were prepared in ultracentrifugation tubes and frozen at −20 °C at an ~45° angle. On the day of purification, a continuous gradient was formed by thawing at room temperature at this angle. Pulse-chase experiments were performed as we described^3^. RPE-J cells transiently over-expressing AcGFP or CIB2-IRES-AcGFP were challenged with ~10 OS/cell in serum-free DMEM with 1 μM MFG-E8 for 1 h at 20 °C (pulse, POS binding only). After washing unbound POS with PBS, cells were chased for indicated times at 32 °C with 5% FBS-supplemented DMEM. At the end of the pulse or chase period, cells were rinsed 3x with PBS before lysis in RIPA buffer with a solution containing 1× protease inhibitors, 1 mM Na orthovanadate, 10 mM Na glycerophosphate, and 10 mM NaF, and stored at −80 °C until immunoblotting.

### X-gal staining, lipid droplet, and indirect lysosome labeling

For X-gal staining, *Cib2*^*WT/WT*^ (control) and *Cib2*^*KO/+*^ enucleated eyes were fixed for 1 h in 0.5% glutaraldehyde (0.5%)+NP-40 (0.02%) diluted in PBS, followed by washing with PBS 2× for 5 min. Eyes were stained overnight at 37 °C in tubes covered with aluminum foil with PBS, in a solution of 1 mg/ml X-Gal, 5 mM K_3_Fe(CN)_6_, 5 mM K_4_Fe(CN)_6_, 2 mM MgCl_2_, 0.02% NP-40, and 0.01% sodium deoxycholate. The next day, the eyes were washed once for 5 min in PBS followed by post-fixation with 2% PFA – 2% glutaraldehyde in PBS for at least 1 h at RT. An opening was made in the lens region to allow OCT penetration and embedding. 20–30 µm thick sections were cut on a Cryotome (Leica, Germany).

Phagosome counts in whole mounts of RPE were performed using Ret-P1 antibody^3^. Whole-mount RPE/choroid preparations were live-stained with 1 µM BODIPY-Pepstatin A or 0.1 mg/ml BODIPY-493/503 in DMEM at 37 °C for 30 min followed by fixation with 4% PFA.

60-70% confluent COS-7 cells or RPE-J cells were fixed with 4% PFA for 15 min at RT and permeabilized with 0.2% Triton X-100 in PBS (15 min), followed by blocking with 10% normal goat serum (NGS) in PBS for at least 30 min at RT. Primary antibodies were diluted in 3% NGS-PBS and incubated overnight at 4 °C, followed by incubations with the indicated goat secondary antibodies. Phalloidin-647 or rhodamine-phalloidin was added at dilutions of 1:1000 (COS-7) or 1:200 (RPE-J) during secondary antibody incubation to stain F-actin. A Zeiss-710 laser scanning confocal microscopy system was used for image acquisition, with a step size of 0.5 µm. Fiji (ImageJ)^71^ was used to process images, and average numbers of phagosomes per 100 µm^2^ of the retina were calculated [detailed in ref. ^30^]. For counting area of undigested material or mitochondria as control (Fig. 2c, d), outlines were drawn in Fiji on TEM images from indicated genotypes of mice sacrificed 8 h after light onset, and areas calculated using Fiji functions.

### Filipin and Oil red O staining

Ten-micron cryosections were prepared using standard procedures and filipin staining for esterified cholesterol (EC) and unesterified cholesterol (UC) was performed^29^. For oil Red O staining, cryosections were dipped in 60% isopropanol for 5 seconds, followed by incubation for 15 min at RT in working solution of Oil Red O (3 parts:2 parts::0.5% Oil Red O solution in isopropanol:water, the working solution was mixed and allowed to stand for 10 min before filtration with vacuum filtration system containing 0.22 µm filter). The slides were then dipped in 60% isopropanol 5 times to remove excess stain and dipped in distilled water 10 times. The imaging was performed with a ×100 objective.

### Cell culture, transfection, and nanoSPD

We optimized the following protocol for 24 wells RPE-J cells transfection using Lipofectamine-2000: Lipofectamine-2000:DNA (2–4 µg plasmid/well)::3:1 was prepared in 100 µl Optimem. Although, the very high plasmid concentration leads to toxicity for most other cell types but works well for RPE-J cells. Cells were used for transfection at ~70% confluence. The medium was replaced with 400 µl Optimem/well (adding Optimem instead of 2% FBS-DMEM is important for transfecting RPE-J cells). The Lipofectamine/plasmid mix was added dropwise. Twenty-four hours later the medium was changed to complete medium (4% FBS-DMEM), and cells were allowed to grow/express plasmid for a further 24 h before fixing or used for pulse-chase experiments, followed by immunoblotting. This method reliably and reproducibly gave us >60% transfection.

A total of 60–70% confluent COS-7 cells in 6-well plates for nanoTRAP were transfected with Lipofectamine-2000 (3:1 ratio) with 1 µg plasmid construct each (nanoTRAP, GFP-tagged bait, and prey). Twenty-four hours after transfection, cells were split 1:10 ratio on glass coverslips to allow for filopodia formation, and fixed 24 h later with 4% PFA for 15 min at RT and permeabilized with 0.2% Triton X-100 in PBS for 15 min at RT, followed by blocking with 10% normal goat serum (NGS) in PBS for at least 30 min at RT. Primary antibodies were diluted in 3% NGS-PBS and incubated overnight at 4 °C, followed by incubations with the indicated goat secondary antibodies. A Zeiss-710 laser scanning confocal microscope or Nikon W1 spinning disk microscope was used for image acquisition, with a step size of 0.5 µm. Fiji (ImageJ)^71^ was used to process images. Finally, LAM-621 and *TSC2*^−^^*/*^^−^, *p53*^−^^*/*^^−^ MEFs were transfected with Lipofectamine-2000 following the manufacturer’s instructions.

### Co-immunoprecipitation, immunoblotting, and mTOR kinase assay

A total of 80% of confluent HEK293 cells in 10-cm dishes were transfected for co-IP experiments with 10 µg of indicated plasmid/s using the polyethylenimine (PEI) method (3:1::PEI:DNA). 2 plates per condition were used. Thirty-six hours later the plates were chilled on ice, washed once with ice-cold PBS, and cells collected in ice-cold PBS and centrifuged for 2 min at 211×*g* at 4 °C. Cell pellet was then lysed in IP buffer (0.3% CHAPS, 40 mM HEPES, 2.5 mM MgCl_2_, 1× Protease inhibitors, 1 mM Na orthovanadate, 10 mM Na glycerophosphate, 10 mM NaF) on ice for 20 min, followed by centrifuging at full speed for 20 min at 4 °C. Lysates were pre-cleared with control agarose beads for 1 h rotating at 4 °C. 30 µl of a 50% slurry of either HA or GST beads washed three times in lysis buffer was added. Samples were rotated 3 h or overnight at 4 °C, washed four times in IP buffer, boiled in 50–100 µl SDS sample buffer at 95 °C, separated by SDS-gel electrophoresis, transferred to PVDF membranes, and immunoblotted.

For GTPγS or GDP loading experiments, cleared lysates of HEK293 cells over-expressing HA-GST-Rheb were prepared as described in IP buffer lacking MgCl_2_. Rheb was immobilized on GST beads by incubating for 2 h, rotating at 4 °C. Beads were washed 4 times with IP buffer without MgCl_2_. GTPγS or GDP was added to final concertation of 100 µM and 1 mM, respectively. The tubes were incubated with shaking for 1 h at 37 °C. Loading was stopped by placing tubes on ice and adding MgCl_2_ to final concentration of 60 mM. TdTomato (control) or tdTomato-CIB2 cleared lysates were prepared as above (in IP buffer) and dividing equally to GTPγS of GDP-loaded Rheb and incubated with rotation overnight. The next day, the beads were washed, processed, and immunoblotted as above. For mTOR kinase assays, *Cib2*^*WT/WT*^ or *Cib2*^*KO/KO*^ animals, were starved for 24 h with regular access to water, to induce mTORC1 activation. The brains were lysed in IP buffer, cleared and mTORC1 was immunoprecipitated using Raptor antibody or rabbit IgG as control. The beads were washed with IP buffer and then kinase buffer twice, and processed as detailed in previously^37^ using 6×His-4E-BP-1.

### Adipose-derived mesenchymal stem cells isolation (MSC), expansion, and culturing

MSCs were isolated as described^72^ with some modifications. Briefly, WT and *Cib2*^*KO/KO*^ (*n* = 3–4/genotype) adipose tissue was dissected from a subcutaneous site of mice at 9 months of age, thoroughly washed with several changes of PBS to remove blood vessels, hairs, and other types of connective tissues, and minced. The minced samples were incubated with the collagenase I solution (10 mg solution in DMEM/3 gm tissue) for 40 min at 37 °C with shaking. After digestion, the sample was diluted 1:1 in culture medium (DMEM supplemented with 15 % FBS and 1×Pen-Strep) and filtered to further disintegrate aggregates. The resulting filtrate was centrifuged at 211×*g* for 10 min, and resulting pellets were re-suspended in 1 ml culture medium and plated the isolated stromal vascular fraction (SVF: consisted of MSC along with circulating blood cells, fibroblasts, pericytes, and endothelial cells) in T-25 culture flasks coated with 1% gelatin. WT and mutant MSCs were grown in 6-well plates on coverslip, for amino acid, insulin, and glucose stimulation experiments, cells were processed as described^37^.

### Retinoid extraction and analysis

All procedures were performed under red safelights. For analysis of mouse ocular retinoids, mouse eye tissues (retinae or eyecups) were homogenized in 1 ml of freshly made hydroxylamine buffer (50 mM MOPS, 10 mM NH2OH, pH 6.5). After the addition of 1 ml ethanol, samples were mixed, then incubated for 30 min in the dark at RT. Retinoids were extracted into hexane (2 × 4 ml) then the solvent was evaporated under a gentle stream of argon at 37 °C. The dried samples were re-dissolved in a 50 μl mobile phase for analysis. Retinaloxime^73^ and retinyl palmitate (Sigma Aldrich, Saint Louis, MO) standards, and samples were separated on a 5 μm LiChrospher Si-60 (ES Industries, West Berlin, NJ) normal-phase column on a Waters H-Class Acquity UPLC (Waters Corp., Milford, MA, USA) in hexane mobile phase containing ethyl acetate (11.2%):dioxane (2.0%):octanol (1.4%) at a flow rate of 1.5 ml/min. Absorbance was monitored at 325 nm for retinyl esters and 350 nm for retinaloximes. Peak areas were integrated and quantified using calibration curves based on external standards. Data were analyzed using Empower 3 software (Waters Corp.).

### A2E extraction and analysis

Pairs of mouse eyes from individual mice were homogenized in 975 µl H_2_O and 2.5 ml methanol–glacial acetic acid (98:2), followed by the addition of 1.125 ml chloroform to get a single-phase solution. The solution was thoroughly mixed and centrifuged for 5 min at 21,130×*g* to pellet proteins. Then 1.225 ml of chloroform and 1.225 ml of H_2_O were added, the tube was mixed for 1 min to separate into two phases and the lower organic phase containing A2E was collected. The solvent was evaporated, and the residue dissolved in acetonitrile (ACN) for analysis. A2E was analyzed by reversed-phase HPLC on a Waters Acquity H-Class UPLC system equipped with an Acquity UPLC BEH C18 column (1.7 μm particles; 2.1 × 50 mm) using isocratic elution with 0.1% trifluoroacetic acid in 20% H_2_O/80% ACN at 1.2 ml/min. Specific wavelength detection was used for monitoring A2E (430 nm), and the quantities of A2E were determined from a calibration curve developed using a synthetic A2E standard. Data were analyzed using Empower 3 software (Waters Corp.).

### Human AMD/ control tissue

Studies on human tissue followed the Declaration of Helsinki guidelines. Human age-matched RPE-choroid tissues were obtained from donor eyes harvested from local and national eye banks, under an IRB exemption issued by the University of Maryland-Baltimore. A complete ocular history, relevant medical history and age was obtained from each donor. Donors with dry AMD and age-matched normal donors were selected for use. We excluded donors with any eye-related diseases besides AMD, or systemic disorders that might affect retinal/RPE or choroidal function. Eyes were examined and RPE/choroidal tissue isolated by a trained Ophthalmologist (SLB) and any additional lesions noted prior to preservation. Tissues were snap-frozen on dry ice and stored at −80 °C prior to use.

### Data analysis

For ERG analysis, four to eight animals were used per time point/genotype/treatment. For in vivo experiments, at least three mouse eyes (from different animals) were used for immunoblots, at least three images were averaged per eye from the three eyes/indicated genotype/time point for confocal microscopy. For cell culture (in vitro) experiments, at least three independent experiments or two independent experiments for co-IP studies were performed. One-way ANOVA with Tukey’s post hoc test or a student’s *t*-test was used to compare the control sample to test samples, with data presented as mean ± SEM. Differences with *p* < 0.05 were considered significant. Data were analyzed using GraphPad Prism (GraphPad Software, Inc., La Jolla, CA). Source data are provided with this paper.

### Statistics and reproducibility

All experiments were performed with at least three animals for in vivo work, and three independent replicates for cell culture-based assays, unless otherwise noted.

### Reporting summary

Further information on research design is available in the Nature Research Reporting Summary linked to this article.

## Supplementary information

Supplementary Information

Reporting Summary

## Data Availability

The data supporting the findings of this study are available from the corresponding author upon reasonable request. Source data are provided with this paper.

## Source data

Source Data
