# Supplementary material for: CIB2 regulates mTORC1 signaling and is essential for autophagy and visual function
Source: Nat Commun. 2021 Jun 23;12:3906. doi: 10.1038/s41467-021-24056-1 (PMC8222345; doi:10.1038/s41467-021-24056-1)
Supplement: Supplementary file 1 — Supplementary Information [file 41467_2021_24056_MOESM1_ESM.pdf]

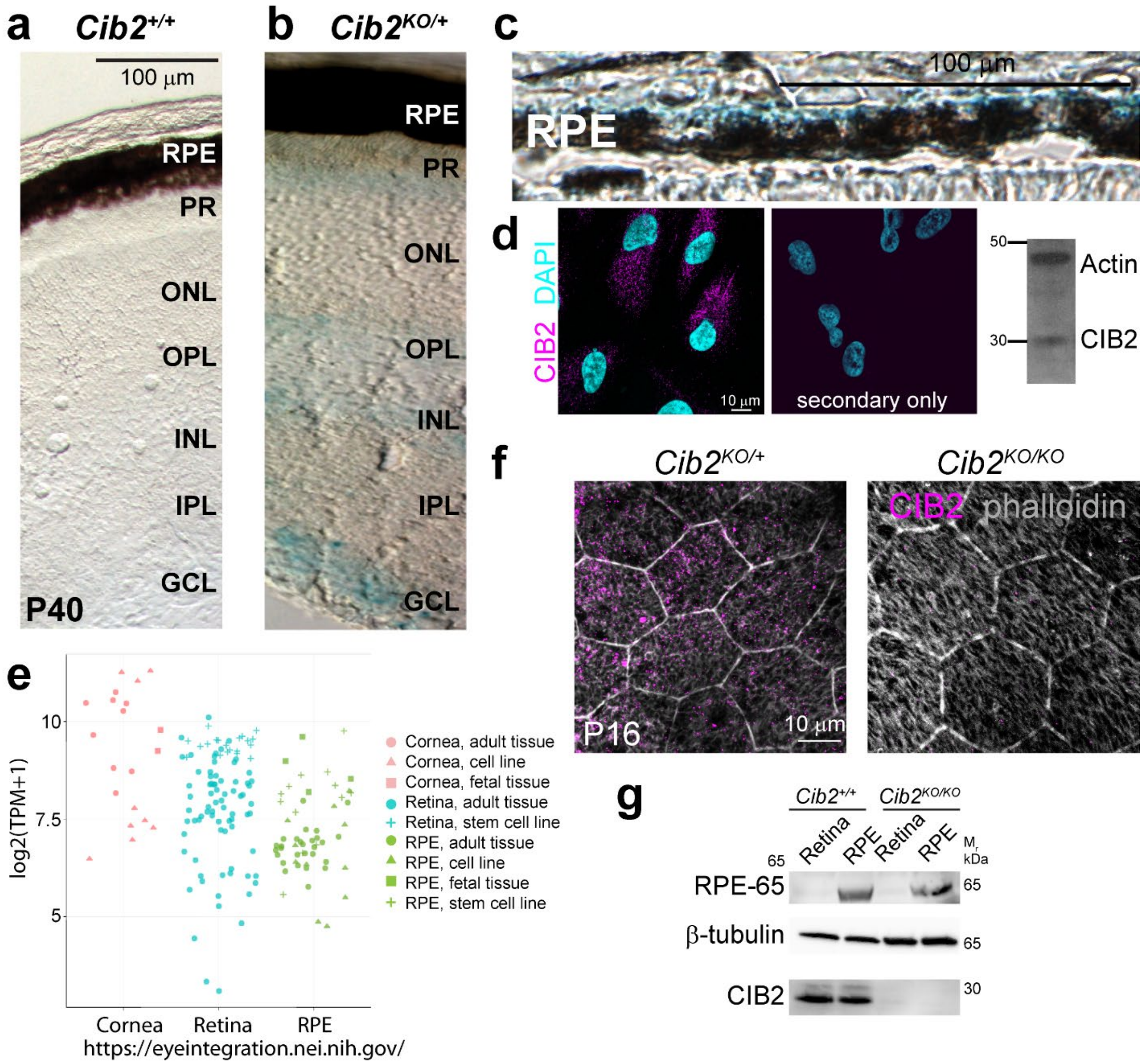

Supplementary Fig. 1: Expression of *Cib2*/CIB2 in the retina

a, b X-gal staining (*blue*) of retina from P40 *Cib2*$^{+/+}$ (**a**) or *Cib2*$^{KO/+}$ (**b**) mouse shows *Cib2* promoter activity in inner segment (IS) of photoreceptors (PR), outer plexiform (OPL), inner nuclear (INL), and ganglion cell layers (GCL). **c** Magnified image of retinal pigment epithelium (RPE) from *Cib2*$^{KO/+}$ mouse stained with X-gal (*blue*) shows *Cib2* promoter activity. **d** CIB2 (*magenta*) immunostaining (*cyan* - DAPI; *left panel*) and immunoblotting (*right panel*) for

indicated proteins from rat RPE-J cell line. **e** Scatter plot of *CIB2* expression in indicated retinal/RPE tissues and cell lines collated by the NEI (https://eyeintegration.nei.nih.gov). **f** CIB2 immunostaining (*magenta*) and actin stained with phalloidin (*grey*) in P16 RPE whole mounts from $Cib2^{KO/+}$ (*left* panel) $Cib2^{KO/KO}$ (*right* panel) shows specific absence of CIB2 staining in mutant mice. ONL-outer nuclear layer. **g** Immunoblot of specified proteins showing lack of CIB2 in the retina and RPE of *Cib2* mutant mice.

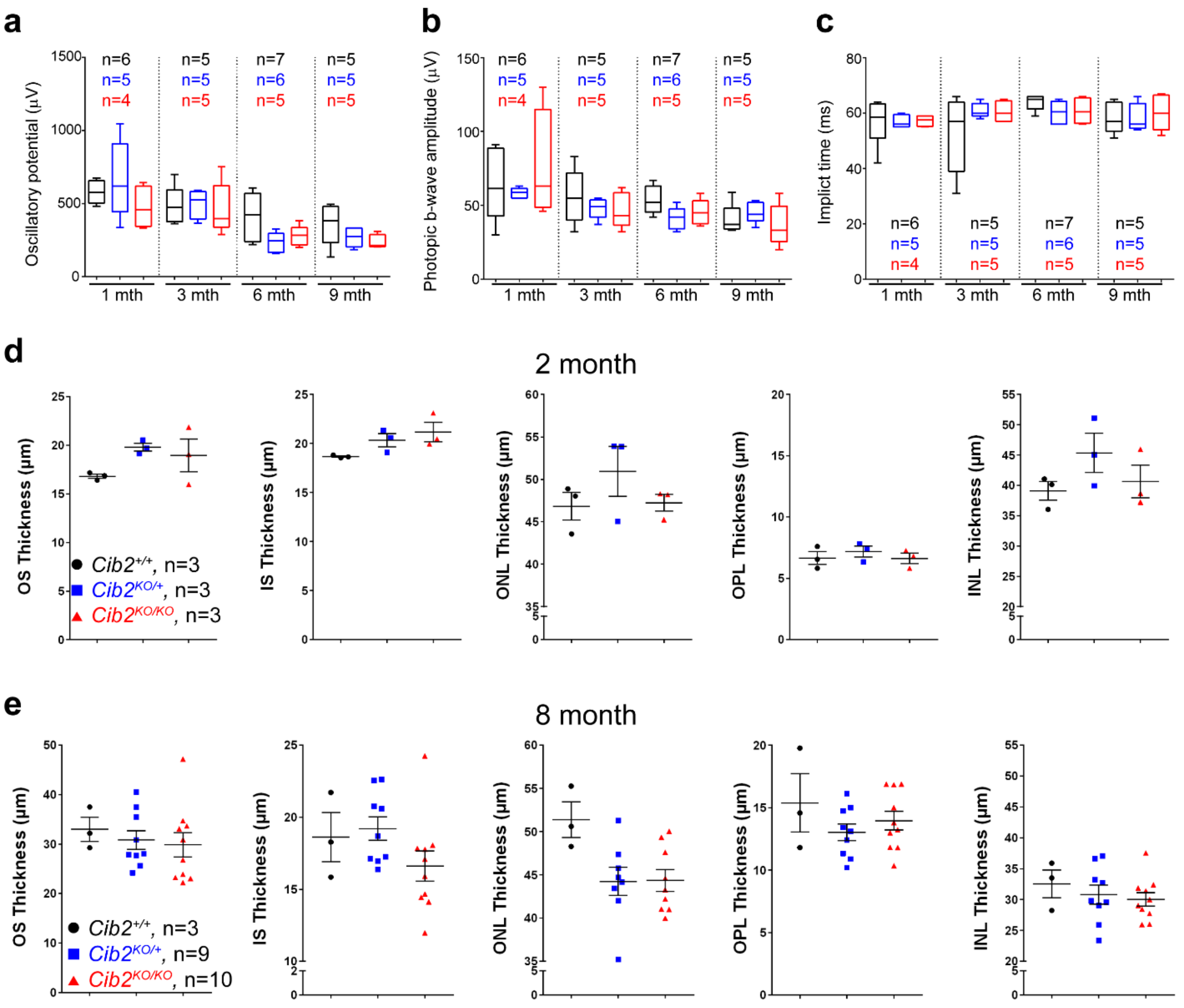

Supplementary Fig. 2: ERG data analyses and retinal morphometry from *Cib2^{KO}* mice

a-c Box and whisker plots for oscillatory potential **(a)**, photopic b-wave **(b)**, and implicit time for scotopic b-wave **(c)** for indicated genotype and time point. n=number of mice for each genotype. Whiskers denote the minimum and maximum measurement for each genotype. **d, e** Morphometric analysis (also see **Methods**) of retinal layer thickness in mice aged 2 months (**d**) or 8 months (**e**) for denoted genotypes as indicated demonstrates no obvious retinal degeneration. OS-outer segments; IS-inner segments; ONL-outer nuclear layer; OPL-outer plexiform layer;

INL-inner plexiform layer. Data presented as mean±SEM; each *point* represents an individual mouse.

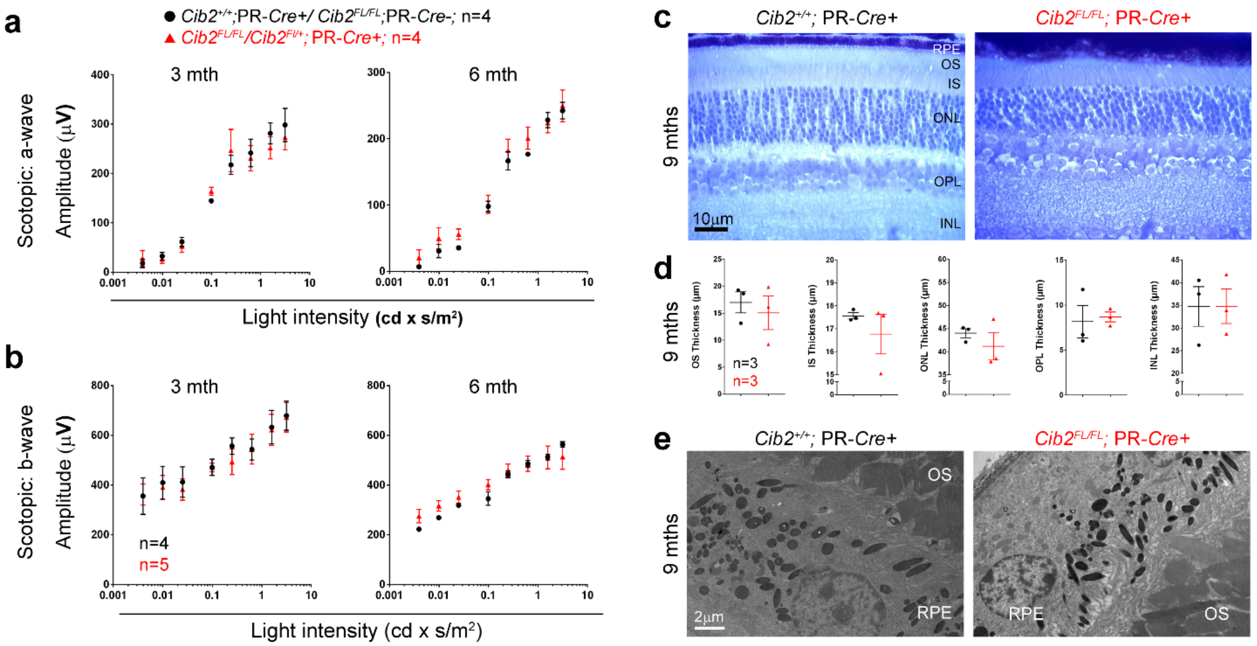

Supplementary Fig. 3: ERG, light microscopic, and TEM analyses of rod photoreceptor-specific *Cib2*^{KO} mice

a, b Quantification of scotopic a- and b-wave amplitudes for indicated genotypes and ages shows no differences in ERG amplitudes in *Cib2* PR mutants when compared to control mice. **c** Light micrographs of indicated genotypes at 9 months of age shows no gross pathology. **d** Morphometric quantification of retinal layers' thicknesses for indicated genotypes at 9 months of age indicates no obvious damage. **e** TEM micrographs of indicated genotypes at 9 months of age showing the interface of the RPE and outer segment (OS) shows no obvious RPE defects. RPE-retinal pigment epithelium; PR-photoreceptors; ONL-outer nuclear layer; OPL-outer plexiform layer; INL-inner plexiform layer; GCL-ganglion cell layer. Data presented as mean±SEM; for **d** each *point* represents an individual animal.

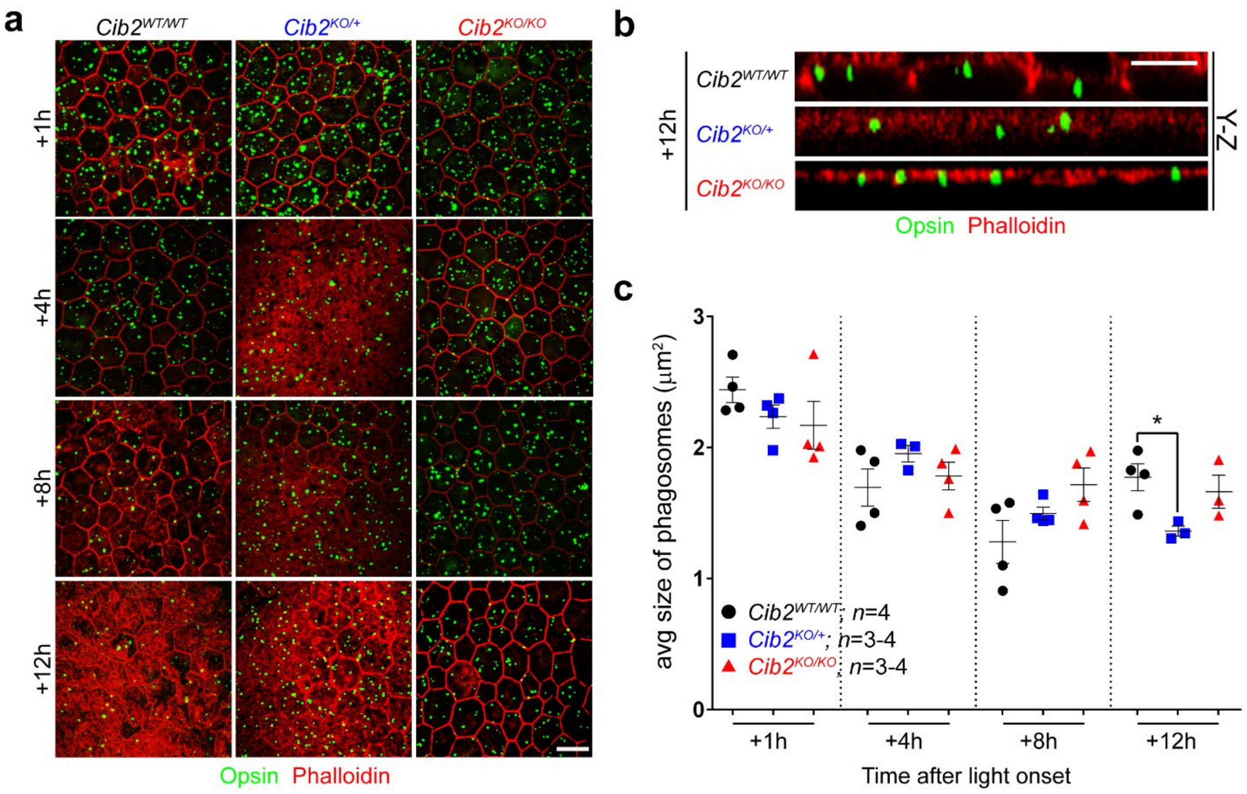

Supplementary Fig. 4: *Cib2^{KO}* mice have aberrant LAP

a Confocal micrographs of flat mount RPE of indicated genotype and times after light onset for 3-4 month old mice show declining opsin-positive phagosome with increasing time elapsed since lights-on. RPE flat mounts are stained with opsin Ret-P1 antibody (*green*) and phalloidin (*red*). In some images the phalloidin covers the entire area of the image showing the intact apical microvilli. **b** Y-Z section from selected areas for the indicated genotypes and time elapsed since lights-on. In RPE of *Cib2^{KO}* mice, the opsin-phagosomes are distributed more apically. **c** Quantification of sizes of opsin-phagosomes with time elapsed indicates that in WT mice the size decreases in concert with time of day, while in mutant mice those changes are much slower, suggesting phagolysosomal digestion defects. *Scale bars*:10 µm. Data presented as mean±SEM; each *point* represents an individual animal. One-way ANOVA and Bonferroni *post hoc* test, $p<0.05$ (*).

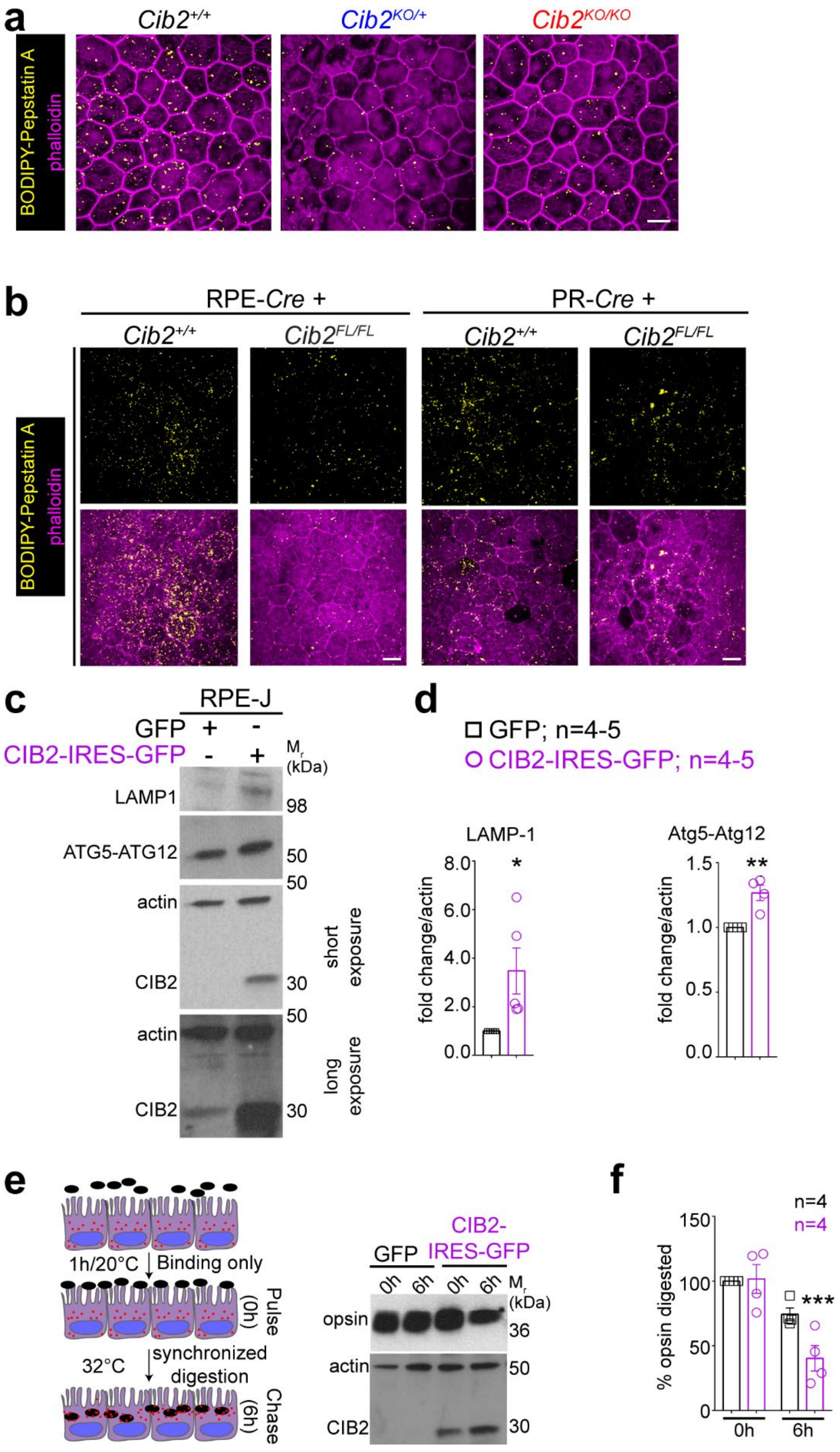
a
Cib2+/+
Cib2KO/+
Cib2KO/KO
BODIPY-Pepstatin A
phalloidin
b
RPE-Cre +
PR-Cre +
Cib2+/+
Cib2FL/FL
Cib2+/+
Cib2FL/FL
BODIPY-Pepstatin A
phalloidin
c
RPE-J
GFP + -
CIB2-IRES-GFP - +
Mr (kDa)
LAMP1
98
ATG5-ATG12
50
50
actin
short exposure
CIB2
30
50
actin
long exposure
CIB2
30
d
GFP; n=4-5
CIB2-IRES-GFP; n=4-5
LAMP-1
Atg5-Atg12
fold change/actin
8.0
6.0
4.0
2.0
0.0
*
1.5
1.0
0.5
0.0
**
e
1h/20°C
Binding only
Pulse (0h)
32°C
synchronized digestion
Chase (6h)
GFP
CIB2-IRES-GFP
0h 6h 0h 6h
Mr (kDa)
opsin
36
actin
50
CIB2
30
f
n=4
n=4
% opsin digested
150
100
50
0
***
0h
6h

Supplementary Fig. 5: *Cib2^{KO}* and RPE-specific but not PR-specific mutant mice have reduced lysosomal capacity, and CIB2 overexpression, *in vitro*, is sufficient to boost levels of lysosomal proteins and OS digestion

a, b RPE whole mounts from 3-4 months old mice of indicated genotype show that global- and RPE-specific, but not PR-specific *Cib2* mutants have less BODIPY-pepstatin A stained lysosomes (related to **Fig. 5a-e**). *Scale bar*, 10 µm. BODIPY-Pepstatin A (*yellow* puncta) and phalloidin (*magenta*). **c, d** Representative immunoblots for indicated autophagy/ lysosomal proteins from RPE-J cells transiently transfected with GFP or CIB2-IRES-GFP and quantified in **d**. Endogenous CIB2 is observable in long exposure immunoblot, while transiently transfected CIB2 is readily observable in short exposure immunoblot. *n* = 5 (LAMP-1) or 4 (ATG5-ATG12) independent transfection experiments. **e, f** Schematic of the pulse-chase phagocytosis assay (*left* panel) – also see **Methods**. Representative opsin immunoblots (*right* panel) for cells pulsed with porcine OS for 1 hr at 20°C (0h) or chased with 5% serum (6h) phagocytosis assay from RPE-J cells transiently transfected with GFP or CIB2-IRES-GFP show that initial binding is unaffected but lower amount of opsin at 6h in CIB2 over-expressing cells suggests faster clearance, quantified in **f**. *n* = 4 independent experiments. Opsin content for RPE cells transiently expressing GFP and pulsed with OS (0h) was set to 100%. Data presented as mean±SEM. Each data point represents an average per individual experiment unless specified. Unpaired two-tailed *t* test (**d, f**), $p<0.05$ (*), <0.01 (**), or <0.001 (***).

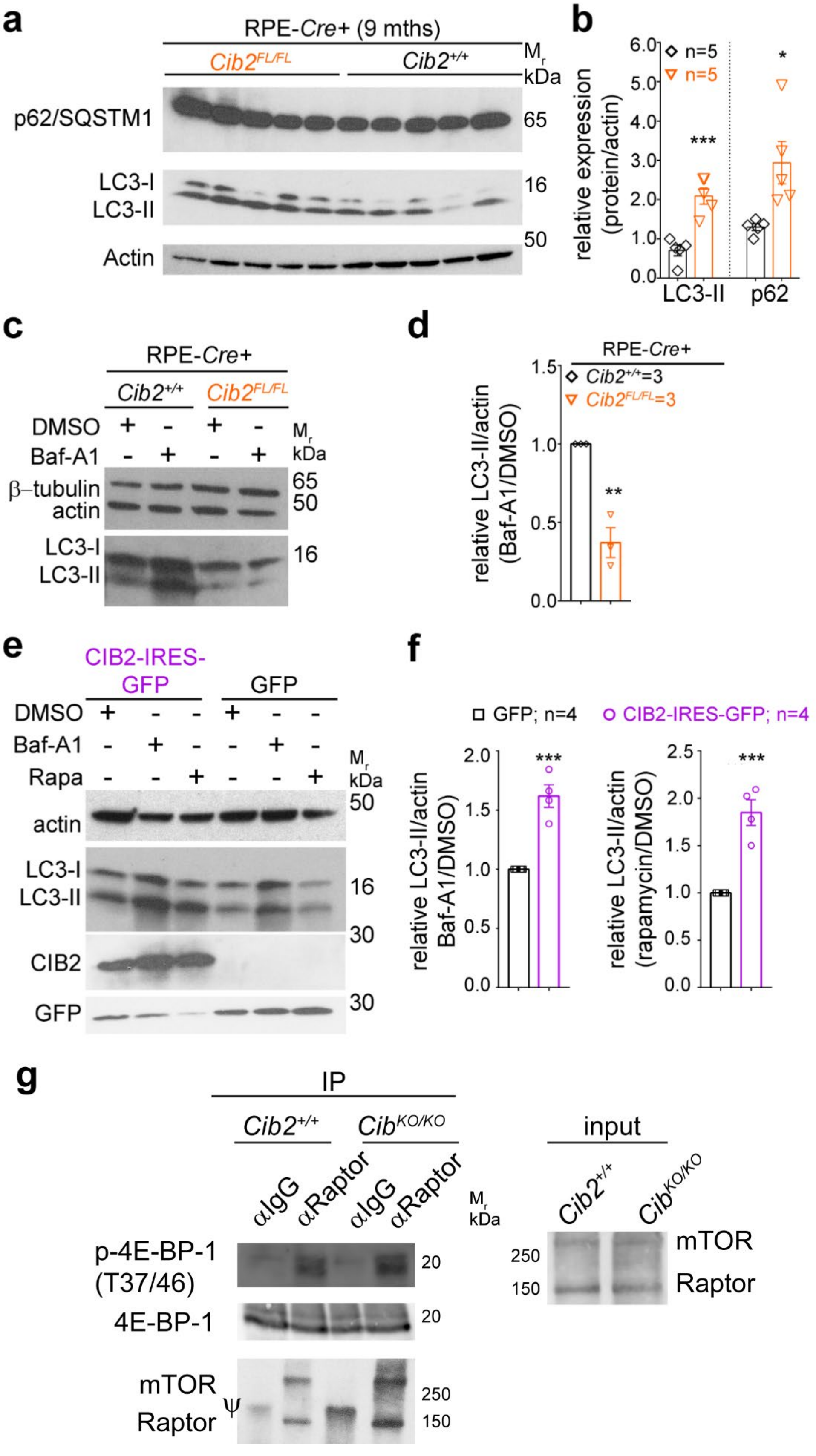
a
RPE-Cre+ (9 mths)
Cib2FL/FL
Cib2+/+
Mr kDa
p62/SQSTM1
65
LC3-I
LC3-II
16
Actin
50
b
n=5
n=5
relative expression (protein/actin)
***
*
LC3-II
p62
c
RPE-Cre+
Cib2+/+
Cib2FL/FL
DMSO
Baf-A1
β-tubulin
actin
65
50
LC3-I
LC3-II
16
d
RPE-Cre+
Cib2+/+=3
Cib2FL/FL=3
relative LC3-II/actin (Baf-A1/DMSO)
**
e
CIB2-IRES-GFP
GFP
DMSO
Baf-A1
Rapa
actin
50
LC3-I
LC3-II
16
30
CIB2
GFP
30
f
GFP; n=4
CIB2-IRES-GFP; n=4
relative LC3-II/actin Baf-A1/DMSO)
relative LC3-II/actin (rapamycin/DMSO)
***
***
g
IP
Cib2+/+
CibKO/KO
αIgG
αRaptor
αIgG
αRaptor
p-4E-BP-1 (T37/46)
20
4E-BP-1
20
mTOR
Raptor
ψ
250
150
input
Cib2+/+
CibKO/KO
mTOR
Raptor
250
150

Supplementary Fig. 6: Loss of CIB2, specifically in the RPE, leads to LC3 flux deficits, while *in vitro* CIB2 overexpression restores LC3 flux

a, b Immunoblots, quantified in **b**, from RPE/ choroid lysates from 9-10 mth old animals of denoted genotype show increased accumulation of LC3-II and p62/SQSTM1, suggesting autophagy deficits at older ages. **c, d** RPE whole mounts treated with DMSO or 50 µM bafilomycin A1 (Baf-A1) overnight show reduced levels of LC3-II, quantified in panel **d**, in RPE-specific *Cib2* deficient mice as compared to controls, indicating autophagy deficits. Ratio for RPE-*Cre*+; *Cib2*$^{+/+}$ was set to 1. **e, f** Representative immunoblots for LC3-II induction in transiently transfected (GFP or CIB2-IRES-GFP) RPE-J cells treated with DMSO, 50 µM bafilomycin-A1 (Baf-A1), or 100 nM rapamycin (Rapa) for 24 hrs, quantified in **f.** Data presented as mean±SEM; each *point* represents an individual animal or experiment. Unpaired two-tailed *t* test (**b, d, f**). p<0.05 (*), <0.01 (**), or <0.001 (***). **g** mTORC1 kinase assays from WT and *Cib2*$^{KO/KO}$ brain lysates shows that immunoprecipitated mTORC1 (using Raptor antibody with IgG as control) phosphorylates 6xHis-4E-BP-1 to much a much greater extent in preps from *Cib2*$^{KO/KO}$ brains, further confirming the mTORC1 hyperactivity in *Cib2*$^{KO/KO}$ mice. $^{\psi}$ non-specific band.

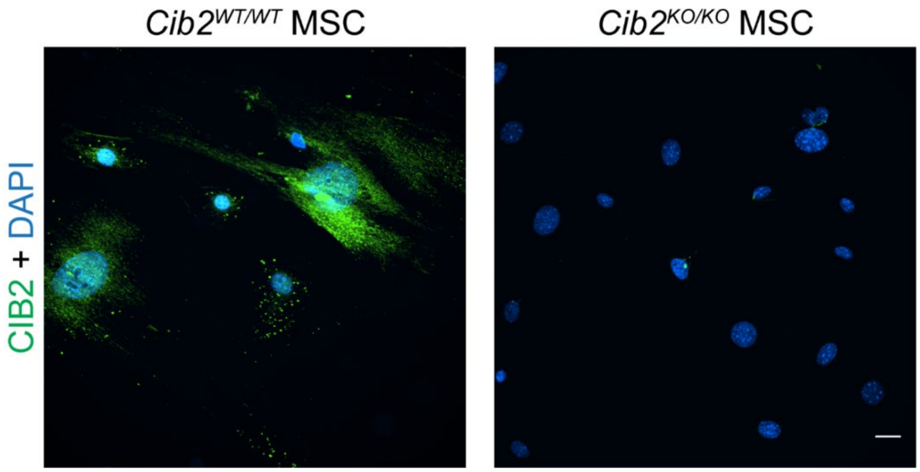

Supplementary Fig. 7: Validation of *Cib2*$^{WT/WT}$ and *Cib2*$^{KO/KO}$ adipose-derived mesenchymal stem cells (MSCs)

MSCs isolated from fat bodies of 9-10 mth old mice of denoted genotype and immunolabeled for CIB2 (green) and DAPI (blue). *Scale bar:* 20 µm.

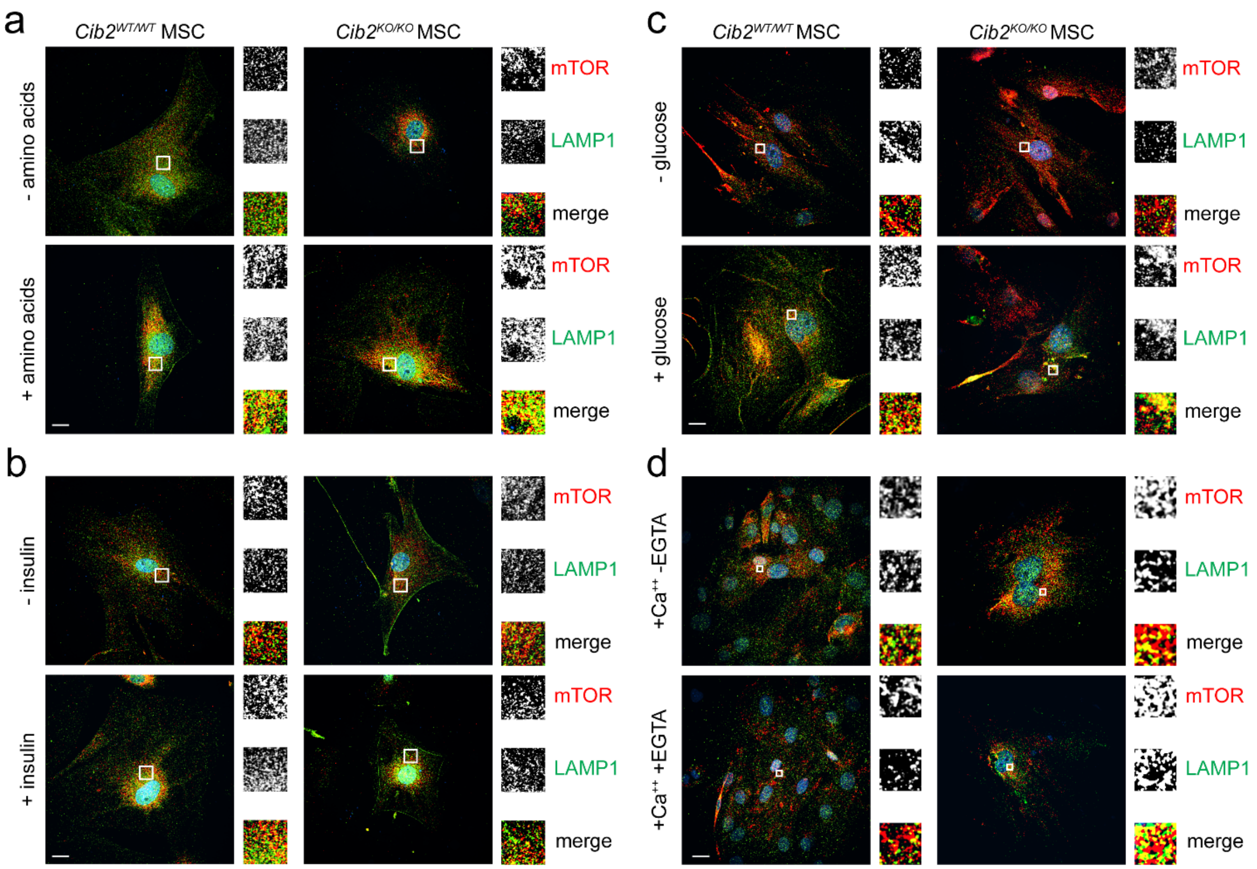

Supplementary Fig. 8: Loss of CIB2 has no apparent impact on the targeting of mTORC1 to lysosomes

a, d Mesenchymal stem cells (MSC) from WT (*left* panels) or *Cib2* mutant mice (*Cib2*$^{KO/KO}$) were **a** serum starved for 16 hrs, followed by amino acid starvation for 40 mins and stimulated with amino acids for 20 mins (+amino acids) or left unstimulated (-amino acids), or **b** serum starved for 40 mins and stimulated with 10 nM insulin (+insulin) or treated with DMEM (-insulin). **c** Cells were glucose starved for 50 mins and restimulated with 50mM glucose (+ glucose) for 10 mins or left unstimulated (- glucose). **d** Cells were placed in HBSS with serum and amino acids and Ca^{++} for 20 minutes and then Ca^{++} was either chelated using EGTA for 5 mins (+ EGTA) or let unchelated (- EGTA). Cells were fixed and stained for lysosomal protein LAMP2 (green) or mTOR (red). Amino acid stimulation lead to co-localization of mTOR with

lysosomes (yellow puncta) irrespective of genotype (**a)** while insulin or glucose stimulation lead to partial co-localization of mTOR with lysosomes (**b, c)**. Ca^{++} chelation lead to greater mTOR/ LAMP1 colocaltion in the absence of CIB2 (**d**). Zoomed in single color images of denoted proteins or merge. *Scale bar:* 20 µm.

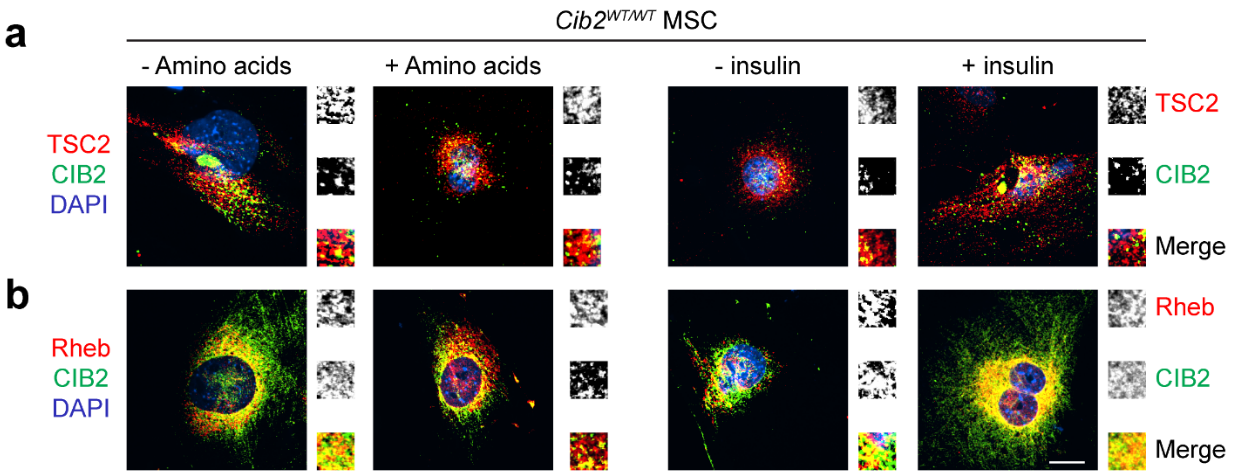

Supplementary Fig. 9: CIB2 co-colocalizes with Rheb but not TSC2

a, b Representative confocal micrographs of denoted proteins in WT mesenchymal stem cells (*Cib2*$^{WT/WT}$ MSC) treated as noted, shows CIB2 (green) doesn't co-localize significantly with TSC (red) **a**, but does with Rheb (red) **b**. DAPI staining (blue) denotes the nuclei. *Scale bar:* 20 µm.

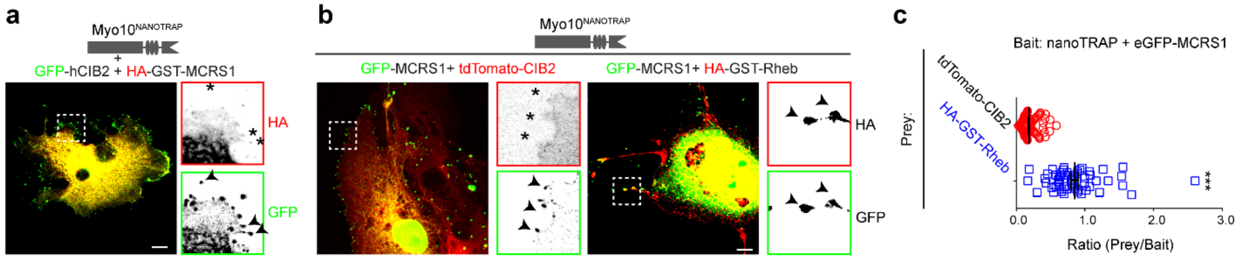

Supplementary Fig. 10: CIB2 doesn't interact with MCRS1

a Merged representative confocal micrographs of bait GFP-hCIB2 (green) with prey HA-GST-MCRS1 (red), and nanoTRAP in COS-7 cells. Zoomed images of inset shows reverse color images of indicated constructs at the tip of filopodia. * Absence at the filopodia tips indicate no interaction of HA-GST-MCRS1 with the GFP-hCIB2. *Arrowheads* indicate accumulation of GFP-CIB2 at the tip of filopodia. **b, c** Merged representative confocal micrographs of bait GFP-MCRS1 (green) with prey CIB2 (red, *left* panel) or Rheb (red, *right* panel) and nanoTRAP in COS-7 cells. Zoomed images of the inset shows reverse color images of indicated constructs at the tip of filopodia. * Absence at the filopodia tips indicate no interaction of CIB2 with the MCRS1 (*left* panel). *Arrowheads* indicate accumulation at the tip of the filopodia and hence interaction of Rheb with MCRS1 (*right* panel). *Scale bar:* 10 µm. The prey/bait ratio is quantified in **c.** Data presented as mean±SEM. Number of filopodia tips analyzed for eGFP-MCRS1 interaction with tdTomato-CIB2 or HA-GST-Rheb is n=90 and n= 57, respectively. At least 3-5 cells for each condition from at least 2 independent experiments were analyzed. Unpaired two-tailed *t* test, $p < 0.001$ (***).

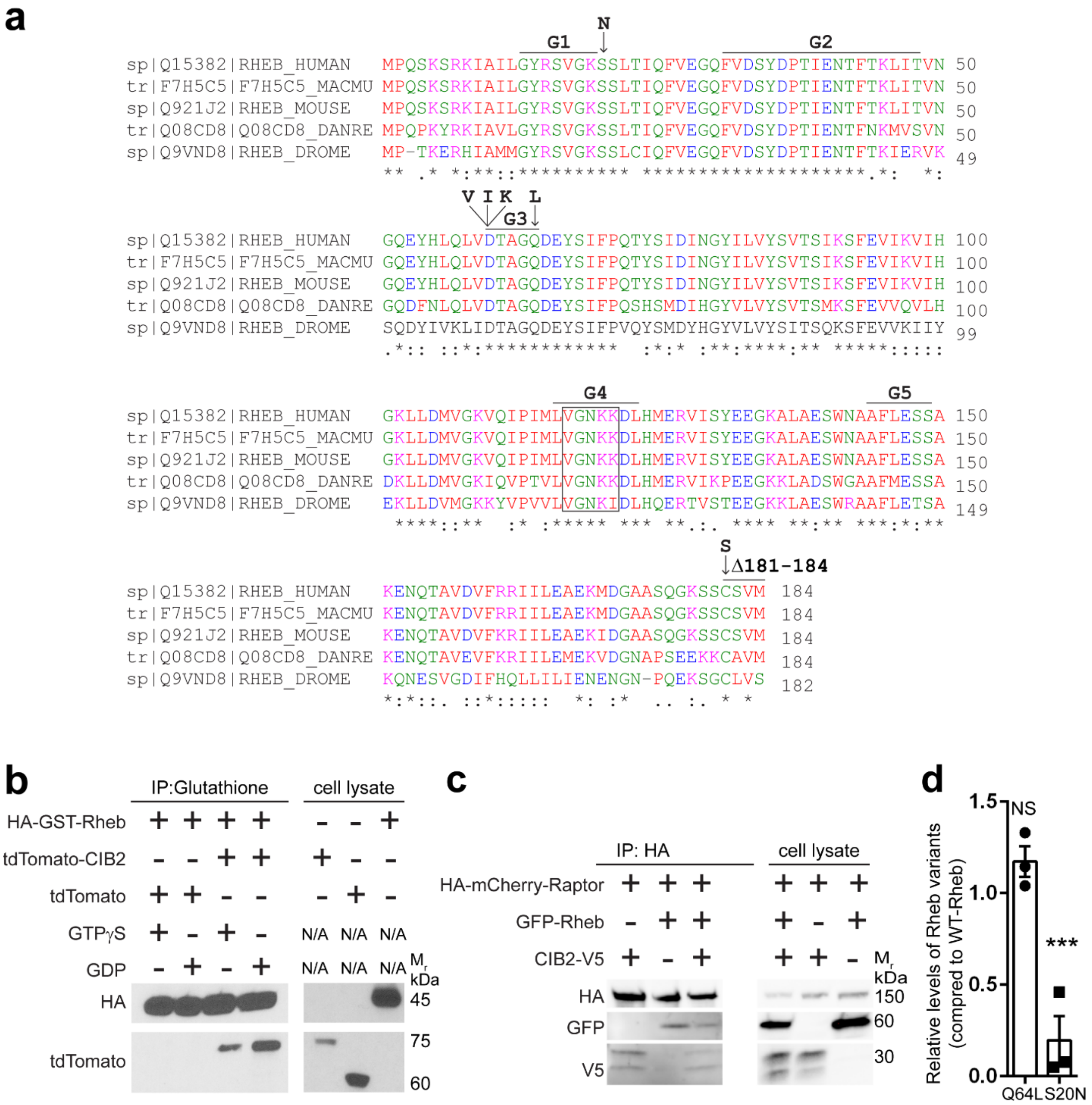

Supplementary Fig. 11: CIB2 interacts with GDP-loaded Rheb and does not alter the interaction of Rheb with Raptor

a Clustal Omega alignment for Rheb from indicated species. The G1-5 loops are indicated along with amino acids substitution variants that were used for co-IP assays in **Fig. 8**. **b** CIB2 preferentially interacts with GDP-loaded Rheb, by co-IP experiments of HA-GST-Rheb

immobilized on GST beads and loaded with either GDP or GTPγS, followed by incubation with tdTomato-CIB2 lysate. **c** co-IP experiments with Rheb and Raptor shows that addition of CIB2 does not impair the interaction of Rheb with Raptor. **d** Immunoblot quantification of Rheb variants p.Q64L and p.S20N, relative to WT-Rheb levels. Data presented as mean±SEM; each *point* represents an individual transfection; n=3. One-way ANOVA and Bonferroni *post hoc* test, $p < 0.001$ (***). NS – not significant.

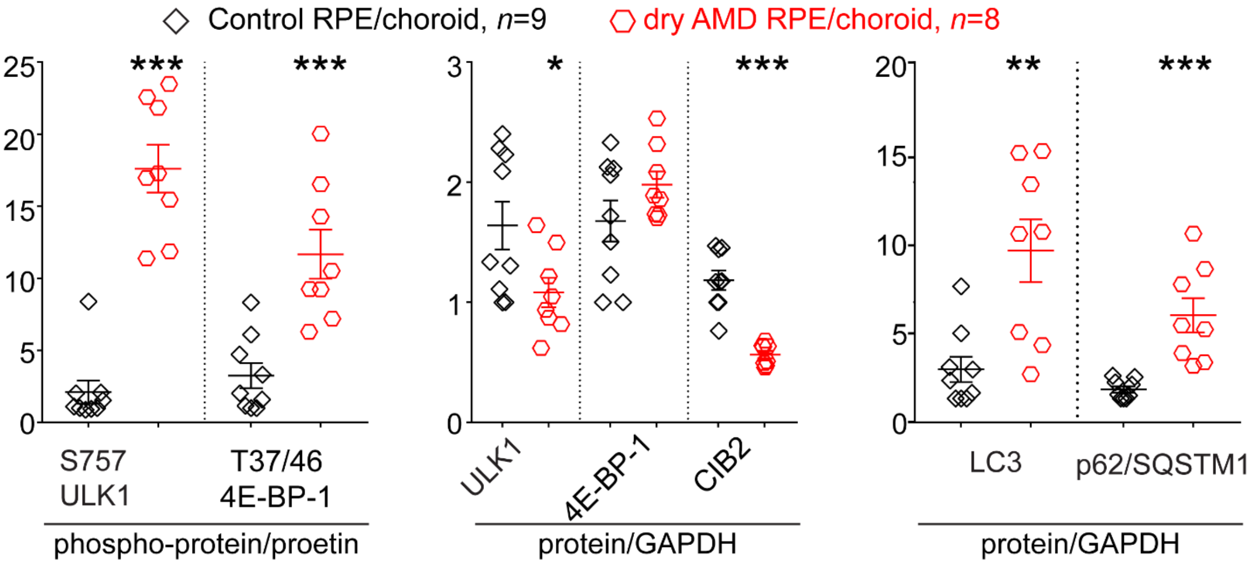

Supplementary Fig. 12: mTORC1 is dysregulated in RPE/choroid lysates from dry AMD cases

Re-quantification of blots shown in **Fig. 9a** with the outlier control sample (**Fig. 9b**, *ψ*) omitted for quantification. Data presented as mean±SEM; each *point* represents an individual donor. Unpaired two-tailed *t* test, *p* <0.05 (*), <0.01 (**), and <0.001 (***)

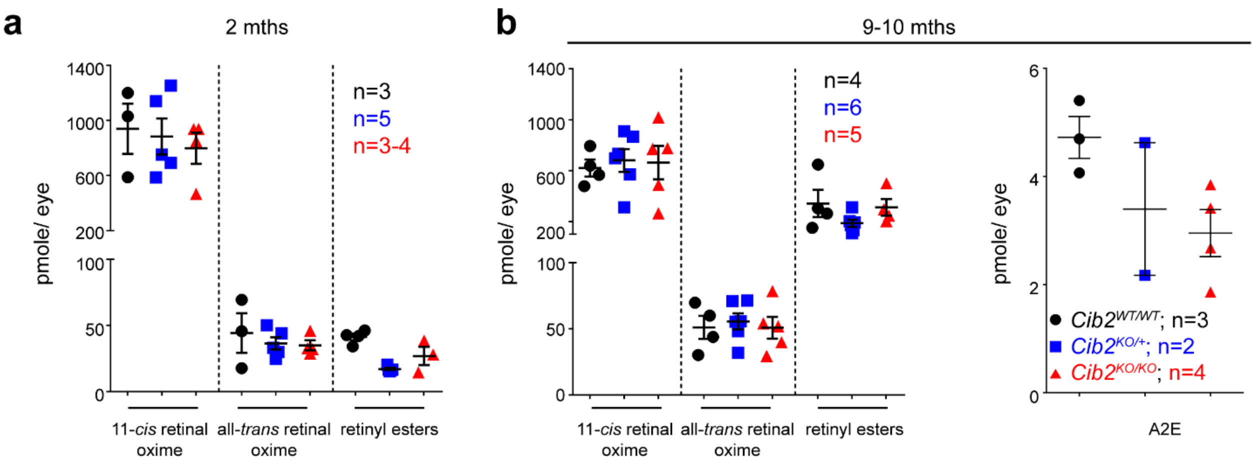

Supplementary Fig. 13: Analysis of retinoid data

a, b Retinoid quantification for indicated retinal derivatives and retinyl esters at ages 2 months **(a)** and 9-10 months **(b)**. Data presented as mean±SEM; each *point* represents an individual animal.

Supplemental Table 1: Deidentified details of the donors for **Fig. 10a, b**.

**Sample #**	**Age (years)**	**Comments**	**Time to preservation**	**Sex**
1	87	Normal	26 hr	female
2	82	Normal/excellent	36hr	male
3	72	Normal	46 hr	male
4	82	Normal	52hr	female
5	70	Normal	44hr	male
6	86	Normal	12hr	female
7	75	Normal	36hr	female
8	80	Normal	14hr	male
9	71	Normal	40hr	male
10	50	Normal	18hr	female
1	80	Hard cuticular drusen in fovea	54hr	female
2	90	Dry AMD	30hr	male
3	68	Drusen in the RPE	38hr	female
4	77	Dry AMD - soft drusen	20hr	male
5	92	Dry AMD	24hr	female
6	85	Dry AMD	30hr	male
7	68	Dry AMD	35hr	female
8	69	Dry AMD	48hrs	female

RPE – retinal pigment epithelium; AMD – age-related macular degeneration.

Supplemental Table 2: Reagents used in the manuscript

REAGENT or RESOURCE	SOURCE	IDENTIFIER
**ANTIBODIES (Anti) (application; dilution)**		
CIB2 (clone 59, homemade) (western; 1:500)	This paper	
Actin (clone 13E5) (western; 1:1000)	Cell Signaling Technology	# 4970S
β-Tubulin (clone 9F3) (western; 1:1000)	Cell Signaling Technology	# 2128S
HA.11 Epitope Tag (western; 1:2000)	Biolegend	# 901501
Cathepsin D (western; 1:500)	R & D systems	AF1029
ATG5; N-terminal (western; 1:5000)	Sigma	A0856
LAMP1 (western; 1:500 for cells, 1: 2000 for tissue)	Sigma	L1418
Rhodopsin Antibody, clone RET-P1 (immunohistochemistry; 1:500)	EMD Millipore	MAB5316
Opsin (clone B6-30); do not boil sample for immunoblot (1:1000)	Novus Biologicals	NBP2-25160
Apolipoprotein E (immunohistochemistry; 1:500)	Abcam	ab183596
C3 (immunohistochemistry; 1:100)	Abcam	ab97462
Beta-amyloid (d3d2n) (immunohistochemistry; 1:100)	Cell Signaling Technology	CST-15126T
LAMP2/ Cd107b (immunohistochemistry; 1:200)	Bio-Rad	RAD-AHP1634
mTOR Substrates Antibody Sampler Kit (western; 1:500 for phospho-proteins, 1:1000 for proteins)	Cell Signaling Technology	# 9862T
SQSTM1/p62 (clone D1Q5S) (western; 1:500)	Cell Signaling Technology	#3 9749
Phospho-AKT 1/2/3 (ser 473) (western; 1:1000)	Santa Cruz Biotechnology	sc-7985
AKT 1/2/3 (western; 1:1000)	Santa Cruz Biotechnology	sc-8312
ULK1 (clone D8H5) (western; 1:1000)	Cell Signaling Technology	# 8054S
Phospho-ULK1 (Ser757) (D7O6U) (western; 1:500)	Cell Signaling Technology	# 14202
GAPDH (clone 0411) (western; 1:1000)	Santa Cruz Biotechnology	sc-47724
LC3B (western; 1:1000)	Novus Biologicals	NB100-2220
DsRed (recognizes tdTomato, clone OTI4C8) (western; 1:500-1000)	OriGene	TA180084
CIB2 (immunohistochemistry; 1:250)	Abnova	H00010518-A01
Myc (clone 9E11) (immunohistochemistry; 1:1000)	Covance	MMS-164P
LAMP1 (immunohistochemistry; 1: 200)	Cell Signaling Technology	# 15665S
Rheb (clone E1G1R) (immunohistochemistry; 1: 200)	Cell Signaling Technology	# 13879S
TUBERIN/TSC2 (immunohistochemistry; 1: 200)	Cell Signaling Technology	# 4308S

RAPTOR	Cell Signaling Technology	# 2280S
**REAGENTS**		
ECL™ Prime Western Blotting System	Thermo Fisher	32106
Lipofectamine™ 2000 Transfection Reagent	Thermo Fisher	11668019
In-Fusion® HD Cloning Plus	Clonetech	638911
BODIPY 493/503	Thermo Fisher	D3922
Pepstatin A, BODIPY™ FL Conjugate	Thermo Fisher	P12271
X-Gal	Sigma-Aldrich	3117073001
9-*cis* retinal	Sigma-Aldrich	R5754-100MG
Filipin Iii	Sigma-Aldrich	SIG-F4767-
Oil red O	Sigma-Aldrich	O0625
Texas Red™-X Phalloidin	Sigma-Aldrich	T7471
Bafilomycin A1	Sigma-Aldrich	B1793
Rapamycin	Sigma-Aldrich	R0395
Pierce™ Anti-HA Agarose	Thermo Fisher	26181
Glutathione Sepharose® 4B	GE Healthcare	GE17-0756-01
Pierce™ Control Agarose Resin	Thermo Fisher	INV-26150
Chaps Hydrate >=98% (hplc)	Sigma-Aldrich	SIG-C3023-25G
Gtp-gamma-s	Abcam	ab146662
Guanosine 5'-diphosphate Sodium Type I	Sigma	G7127
QuikChange Lightning Site-Directed Mutagenesis Kit	Agilent	210518
Accutase	Sigma-Aldrich	SIG-A6964
ProBond Purification system	Invitrogen	45-0055
**Recombinant Proteins**		
Recombinant Human MFG-E8 Protein	R & D systems	2767-MF-050
4EBP1 Protein, Human, Recombinant (His Tag)	Sino Biological US Inc.	10022-H07E
**Experimental Models: Cell Lines**		
RPE-J	ATCC	# CRL-2240
HEK-293	ATCC	# CRL-1573
LAM-621	Kind gift from Dr. Henske	Yu et al., 2004
Tsc2$^{-/-}$, *p53*$^{-/-}$ MEF		Zhang et al., 2003
**Recombinant DNA**		
pAAV-CIB2-IRES-AcGFP	This paper	
pAAV-AcGFP	Grousbeck Gene Therapy Center	
pRK5-HA-mCherry-raptor	Addgene	73386
pRK5-HA-GST-Rheb1	Addgene	19310

pRK5-myc-mTOR	Addgene	1861
pRK7-FLAG-TSC1	Addgene	8995
pRK7-FLAG-TSC2	Addgene	8996
eGFP-C2-hCIB2	This paper	
tdTomato-C1-mCIB2	This paper	
ptdTomato Vector-C1	Clonetech	632531
pcDNA3.1-MYO10-HMM-Nanotrap	Addgene	87255
eGFP-C2-hFAM92A1		Schrauwen et al, 2018
eGFP-C2-hMETTL5		Richard et al, 2019
Human Mcrs1	Sino Biological US Inc.	SNB-HG14816-G
**Software and Algorithms**		
EM for Windows	LKC	
Prism 6.0	GraphPad, La Jolla, CA	
Adobe Suite	Adobe, CA	
Fiji	https://fiji.sc/	
**Mouse strains**		
Cib2$^{tm1a(EUCOMM)Wtsi}$ mice	EUCOMM	EM:05417
VMD2-Cre mice	Gift from Drs. Sheldon Miller and Joshua Dunaief	Iacovelli et al., 2011
Opsin–iCre75	Jax	Stock No: 015850
ROSA26::FlPe knock in mice	Jax	Stock No: 003946
