# Supplementary material for: CIB2 regulates mTORC1 signaling and is essential for autophagy and visual function
Source: Nat Commun. 2021 Jun 23;12:3906. doi: 10.1038/s41467-021-24056-1 (PMC8222345; doi:10.1038/s41467-021-24056-1)
Supplement: Supplementary file 2 — Reporting Summary [file 41467_2021_24056_MOESM2_ESM.pdf]

nature research

Corresponding author(s): Zubair M. Ahmed, PhD

Last updated by author(s): May 25, 2021

Reporting Summary

Nature Research wishes to improve the reproducibility of the work that we publish. This form provides structure for consistency and transparency in reporting. For further information on Nature Research policies, see our Editorial Policies and the Editorial Policy Checklist.

Statistics

For all statistical analyses, confirm that the following items are present in the figure legend, table legend, main text, or Methods section.

n/a	Confirmed	
☐	☒	The exact sample size (*n*) for each experimental group/condition, given as a discrete number and unit of measurement
☐	☒	A statement on whether measurements were taken from distinct samples or whether the same sample was measured repeatedly
☐	☒	The statistical test(s) used AND whether they are one- or two-sided<br>*Only common tests should be described solely by name; describe more complex techniques in the Methods section.*
☒	☐	A description of all covariates tested
☐	☒	A description of any assumptions or corrections, such as tests of normality and adjustment for multiple comparisons
☐	☒	A full description of the statistical parameters including central tendency (e.g. means) or other basic estimates (e.g. regression coefficient) AND variation (e.g. standard deviation) or associated estimates of uncertainty (e.g. confidence intervals)
☐	☒	For null hypothesis testing, the test statistic (e.g. F, t, r) with confidence intervals, effect sizes, degrees of freedom and P value noted<br>*Give P values as exact values whenever suitable.*
☒	☐	For Bayesian analysis, information on the choice of priors and Markov chain Monte Carlo settings
☒	☐	For hierarchical and complex designs, identification of the appropriate level for tests and full reporting of outcomes
☒	☐	Estimates of effect sizes (e.g. Cohen's d, Pearson's r), indicating how they were calculated

Our web collection on statistics for biologists contains articles on many of the points above.

Software and code

Policy information about availability of computer code

Data collection	N/A
Data analysis	N/A

For manuscripts utilizing custom algorithms or software that are central to the research but not yet described in published literature, software must be made available to editors and reviewers. We strongly encourage code deposition in a community repository (e.g. GitHub). See the Nature Research guidelines for submitting code & software for further information.

Data

Policy information about availability of data

All manuscripts must include a data availability statement. This statement should provide the following information, where applicable:

- Accession codes, unique identifiers, or web links for publicly available datasets
- A list of figures that have associated raw data
- A description of any restrictions on data availability

All data are described in the manuscript

Field-specific reporting

Please select the one below that is the best fit for your research. If you are not sure, read the appropriate sections before making your selection.

☒ Life sciences ☐ Behavioural & social sciences ☐ Ecological, evolutionary & environmental sciences

For a reference copy of the document with all sections, see nature.com/documents/nr-reporting-summary-flat.pdf

Life sciences study design

All studies must disclose on these points even when the disclosure is negative.

Sample size	Sample size (n) is supplied in figure or legends in main paper or the supporting information file. All sample size was determined by number of the biological replicate necessary to ensure statistical significance, so as to support meaning conclusions, and based on the sample number from preliminary studies and similar studies (e,g, Giese et al, Nat Commu, 2017.).
Data exclusions	no data was excluded
Replication	Certain key experiments were reproduced with independent cohort in a different collaborator's lab.
Randomization	males and females were both used for experiments and were randomized to control/treatment group as the case maybe.
Blinding	for relevant experiments, the person performing the experiment or the analyses was blinded and data was unblinded after analysis.

Reporting for specific materials, systems and methods

We require information from authors about some types of materials, experimental systems and methods used in many studies. Here, indicate whether each material, system or method listed is relevant to your study. If you are not sure if a list item applies to your research, read the appropriate section before selecting a response.

Materials & experimental systems

n/a	Involved in the study	
☐	☒	Antibodies
☐	☒	Eukaryotic cell lines
☒	☐	Palaeontology and archaeology
☐	☒	Animals and other organisms
☐	☒	Human research participants
☒	☐	Clinical data
☒	☐	Dual use research of concern

Methods

n/a	Involved in the study	
☒	☐	ChIP-seq
☒	☐	Flow cytometry
☒	☐	MRI-based neuroimaging

Antibodies

Antibodies used	Rabbit antibody clone 59 for CIB2 was generated by Covance
Validation	Our custom made Clone 59 Ab has been validated in Cib2 knockout mice - Supp fig 1g

Eukaryotic cell lines

Policy information about cell lines

Cell line source(s)	ATCC
Authentication	Cell lines were received fresh from ATCC
Mycoplasma contamination	all cell lines tested negative for mycoplasma
Commonly misidentified lines (See ICLAC register)	No commonly misidentified cell lines were used.

Animals and other organisms

Policy information about studies involving animals; ARRIVE guidelines recommended for reporting animal research

Laboratory animals	Animal strains are described in detail in the manuscript, along with relevant references
Wild animals	N/A
Field-collected samples	N/A
Ethics oversight	All animal procedures were reviewed and approved by the IACUC (Institutional Animal Care and Use Committees) of the University of Maryland School of Medicine

Note that full information on the approval of the study protocol must also be provided in the manuscript.

Human research participants

Policy information about studies involving human research participants

Population characteristics	Studies on human tissue followed the Declaration of Helsinki guidelines. Human age-matched RPE-choroid tissues were obtained from donor eyes harvested from local and national eye banks, under an IRB exemption issued by the University of Maryland-Baltimore. A complete ocular history, relevant medical history and age was obtained from each donor. Donors with dry AMD and age-matched normal donors were selected for use. We excluded donors with any eye-related diseases besides AMD, or systemic disorders that might affect retinal/RPE or choroidal function. Eyes were examined and RPE/choroidal tissue isolated by a trained Ophthalmologist (SLB) and any additional lesions noted prior to preservation
Recruitment	N/A
Ethics oversight	N/A

Note that full information on the approval of the study protocol must also be provided in the manuscript.
